# An Energy-Aware Hybrid ARQ Scheme with Multi-ACKs for Data Sensing Wireless Sensor Networks

**DOI:** 10.3390/s17061366

**Published:** 2017-06-12

**Authors:** Jinhuan Zhang, Jun Long

**Affiliations:** School of Information Science and Engineering, Central South University, Changsha 410083, China; jinhuan_zhang@csu.edu.cn

**Keywords:** wireless sensor networks, energy efficiency, reliable communication, energy consumption, multiple ACK copies

## Abstract

Wireless sensor networks (WSNs) are one of the important supporting technologies of edge computing. In WSNs, reliable communications are essential for most applications due to the unreliability of wireless links. In addition, network lifetime is also an important performance metric and needs to be considered in many WSN studies. In the paper, an energy-aware hybrid Automatic Repeat-reQuest protocol (ARQ) scheme is proposed to ensure energy efficiency under the guarantee of network transmission reliability. In the scheme, the source node sends data packets continuously with the correct window size and it does not need to wait for the acknowledgement (ACK) confirmation for each data packet. When the destination receives *K* data packets, it will return multiple copies of one ACK for confirmation to avoid ACK packet loss. The energy consumption of each node in flat circle network applying the proposed scheme is statistical analyzed and the cases under which it is more energy efficiency than the original scheme is discussed. Moreover, how to select parameters of the scheme is addressed to extend the network lifetime under the constraint of the network reliability. In addition, the energy efficiency of the proposed schemes is evaluated. Simulation results are presented to demonstrate that a node energy consumption reduction could be gained and the network lifetime is prolonged.

## 1. Introduction

Edge computing covers a wide range of technologies and wireless sensor networks (WSNs) are one of its important supporting technologies. WSNs are widely applied in more and more fields, such as the Internet of Things, Cyber Physical Systems, Vehicle Systems and Smart Cities [1,2,3,4,5,6,7]. Because sensor nodes have limited batteries and these are hard to replace, the network lifetime is an important performance metric that needs to be considered in any WSN study [8,9,10,11,12]. In addition, due to transmission interference, obstacles and intrusions, wireless links are unreliable. Thus, many errors occur in the data packet transmission process. To ensure the transmission reliability is a key issue for successful application of WSNs. However, ensuring 100% reliable transmission is costly and unnecessary in some wireless communication cases. For example, many applications such as environment (temperature, humidity), or agriculture (water tank, irrigation) monitoring require that each data packet be successfully delivered to the sink with a statistical probability δ < 1, such as 60%–95% [13].

Moreover, how to prolong the network lifetime with the assurance of transmission reliability is a challenging issue. Among the two reliable transmission schemes—packet-loss avoidance and packet-loss recovery [13]—since the packet-loss avoidance is costly, reliable protocols based on packet-loss recovery are more widely applied in WSNs [14,15,16]. However, in a typical packet-loss recovery scheme (e.g., Automatic Repeat-reQuest (ARQ)), when any one packet runs into a transmission error, it is recovered by retransmitting the packet. To reduce the number of retransmissions for packet loss recovery and improve the energy efficiency, the idea of using multiple acknowledgements (ACKs) is proposed. Nguyen et al. [17] proposed a multi-ACK data forwarding scheme to improve the reliability and energy efficiency of WSNs. However, this work just gave a simple analysis of energy consumption and transmission delay for individual nodes. Liu et al. [18] proposed the protocol named send and wait one data multi-ACK (SW-ODMA) ARQ protocol to improve the energy efficiency and statistical reliability. In the scheme, the transmitter just sends one data packet and multiple ACKs are returned from the receiver after receiving the data packet. Dong et al. [19] proposed a novel data gathering protocol named Broadcasting Combined with Multi-Negative Acknowledgement (NACK)/Acknowledgement (ACK) (BCMN/A) based on the analysis strategy to meet requirements of a sensing application through trade-offs between the energy consumption and source-to-sink transport delay under the reliability constraint of wireless sensor networks. In the scheme, the data gathering deploys the send and wait hop-by-hop automatic repeat-reQuest (SW HBH ARQ) protocol. Accordingly, the data load analysis and energy consumption are also under SW HBH ARQ protocol control.

To ensure the data packets are transmitted continuously without waiting for confirming messages, the hybrid ARQ scheme to enable source nodes to send data packets continuously with the window size of *W* is considered [20]. Accordingly, an energy-aware hybrid ARQ scheme for unicast is proposed to ensure energy efficiency and transmission reliability in this paper. Compared with the previous study, we make the following contributions:

(1) The energy-aware hybrid ARQ scheme for unicast is proposed to ensure the energy efficiency under the guarantee of network transmission reliability. In the scheme, the source node sends data packets continuously with the window size of *W* and it is not necessary to wait for the confirming ACK of each packet. When the sink receives *K* data packets, it will return multiple copies of one ACK for confirmation. After the source node has received ACK packets with some statistical success probability satisfying the transmission constraint, it would send the subsequent packets the window size of *W*. Otherwise, the source node would retransmit the data packets in the same window.
(2) The energy cost of the proposed scheme is statistically analyzed for each node in a flat circle network. Under the typical reliable data transmission model and applying the statistical theory, the theoretical analysis on data load of each node in the flat circle network is presented to ensure the constraint of network transmission reliability is met. Furthermore, the energy cost of each node in one round of data gathering is statistically analyzed based on the classical energy consumption model.
(3) The conditions under which the proposed energy-aware hybrid ARQ can be more effective than the original ARQ scheme are discussed. In addition, based on the analysis of the trade-off between node data load and the network statistical reliability, how to select parameters including the transmission range *r*, the number of returned copies of ACK n for *K* packets received is described considering the constraint of required reliability δ to prolong the network lifetime.

The rest of this paper is organized as follows: Section 2 reviews related works. The system model is described in Section 3. The energy-aware hybrid ARQ scheme and its theoretical energy cost analysis are demonstrated in Section 4, including the analysis and discussions on energy-efficiency conditions and how to select the optimized parameters to achieve the optimal goal of prolonging the network service lifetime. Section 5 presents the simulation results to evaluate the efficiency of the proposed ARQ scheme. We conclude in Section 6.

## 2. Related Work

There are a great deal of research work on WSNs, including reducing end-to-end delay [21,22,23,24], improving energy efficiency and transmission reliability [4,8,9,10,11,12,13,14], and other aspects [3,5,6,7,25]. Focusing on the transmission characteristics of WSN, the research on energy-efficient and reliable data transmission theory and application technology is of great significance [19,20]. However how to reduce the energy cost with the guarantee of the transmission reliability is a challenging issue. To ensure reliable communication is essential for most wireless sensor network applications. Due to the unreliability of links, a great deal of research efforts have been devoted to this field to provide reliable transmission service in WSNs. Liu et al. [13] pointed out that the existing works mainly fall into two categories: packet-loss avoidance and packet-loss recovery. The packet-loss avoidance approach (e.g., [26,27]) attempts to reduce the occurrence of packet loss and the packet-loss recovery (e.g., [8,15,16,28]) tries to recover from packet loss when it happens. Because packet-loss avoidance methods are costly, due to cost considerations the most widely used mechanisms in networks are based on packet-loss recovery. One type of reliable protocol based on packet-loss recovery is the retransmission after packet loss and its representative protocol is Automatic Repeat-reQuest (ARQ). 

Rosberg et al. [20] introduced a new notion of statistical reliability to achieve a balance between data reliability and energy consumption. Based on this, the energy efficiency of a comprehensive set of statistically reliable data delivery protocols are analyzed for linear networks. Liu et al. [18] studied the protocol named send and wait one data multi-ACK (SW-ODMA) ARQ protocol to improve the energy efficiency and statistical reliability. In the protocol, the transmitter sends one data packet at a time and multiple ACKs are returned after receiving the data packet at the receiver. When the returned ACKs are received, the transmitter will send the next data packet. Through reducing the returned ACKs loss, this achieves the goal of reducing retransmission times for sending data packets that consumes more energy. However, in the SW-ODMA scheme, the transmitter will not send the next packet until it receives ACK confirming package or it will resend the last packet when there is a timeout. For coping with the harsh conditions where link packet error rate may be as high as 70% and path length could be up to tens of hops, Jung et al. [29] proposed the hybrid automatic repeat request (HARQ) scheme for wireless sensor network multicast services. In the proposed algorithm, the HARQ operation is combined with an autonomous retransmission method that ensures a data packet is transmitted irrespective of whether or not the packet is successfully decoded at the receiver. However, the traditional per-hop and end-to-end (E2E) recovery schemes suffer from low E2E success rate and poor energy efficiency in large-scale real environments. To address these problems, Liu et al. [13] introduced a novel in-middle recovery scheme and realize it by designing and implementing a proliferation routing, which integrates three core technologies, namely, capability-based path finder, a randomized dispersity, and reproduction. Proliferation routing (PR) offers great flexibilities for transmissions. It can not only be applied with any Medium Access Control (MAC) protocol and routing metric, but also obtains a desired service quality (i.e., transmission success rate, energy cost, etc.) by controlling the system parameters. Although a PR scheme ensures transmission success by packet reproduction in the transmission process to the destination, it cannot necessarily improve the network lifetime effectively. More reproduced data packets will lead to more energy cost for transmission to the destination. The sensors close to the sink deplete their energy faster than other sensors because these sensors have to relay many more data packets for a large part of the network. 

Therefore, to reduce the number of retransmissions for packet loss recovery is a more effective approach to decrease the network energy consumption and extend the network lifetime for ARQ. Moreover, in order to ensure the data packets are transmitted continuously and without waiting for the confirmation messages of each successful transmitted data packet, an energy-aware hybrid ARQ scheme for unicast is proposed in the paper to address these issues. 

## 3. The System Model and Problem Statement

### 3.1. The System Model

Consider a wireless sensor network consisting of sensor nodes that are randomly scattered in a flat region with the radius of *R* (m). Nodes do not move after being deployed to detect the event. The sensor node periodically generates a message contained in multi-packets, and forwards the packets with a window size of *W* to a special destination node, called the sink. The sink is located at the center point of the network. Assume the information generated at each sensor node is exact. In one round of data gathering, each sensor node not only generates and transmits its own sensed data packets, but also relays messages from other nodes to the sink via multi-hop wireless communications. The message routing is fixed and the time is slotted. All sensor nodes are assumed to be synchronized. Moreover, each node has an identical transmission range of *r* (m). 

**Figure 1:**
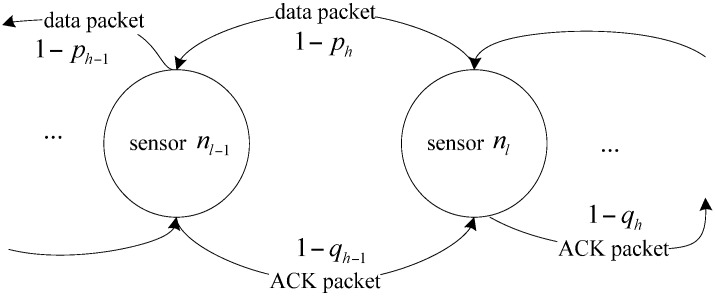
Reliable transmission model.

The wireless channel is assumed with nonzero packet loss reliability. A packet loss can be restored by retransmitting the lost packet, which, however, results in additional energy cost. Assuming a node *n_l_* has the distance *l* = *ir* + *x* (m) to the sink, then the probability of a data packet being successfully transmitted from node *n_l_* to other nodes on the routing path is denoted as 1 − *p_i_*, and 1 − *q_i_* denotes the successful transmission probability of the returned ACK packet. The reliable transmission model is shown in Figure 1 [20]. For simplification, the probability ${\bar{p}}_{i}$ denotes 1 − *p_i_* and ${\bar{q}}_{i}$ denotes 1 − *q_i_*. Besides, the system model assumes that the successful transmission probabilities of data or ACK in one hop transmission are the same for all links. Because of the unreliable links, some packets may need to be retransmitted to ensure the network reliability. In our application, the network statistical reliability is set to be *δ* (0 < *δ* < 1) from one source node to the sink. 

### 3.2. Energy Consumption Model

Following the typical energy consumption model in [30], the expected energy cost for transmitting and receiving a packet is computed by:

(1) $$\left\{ \begin{array}{l} E_{T}=l_{p}E_{elec}+l_{p}\varepsilon_{fs}d^{2}\;if(d<d_{0})\\ E_{T}=l_{p}E_{elec}+l_{p}\varepsilon_{amp}d^{4}\;if(d>d_{0})\\ E_{R}(l_{p})=l_{p}E_{elec} \end{array}\right.$$

where, *E_elec_* denotes the transmitting circuit loss energy and *d*_0_ denotes the threshold. The parameters *ε_fs_* and *ε_amp_* respectively represent the energy required by the power amplifier and *l_p_* indicates the packet length (bits). Following the parameters in [30], the network energy consumption parameters and the corresponding values are shown in Table 1.

### 3.3. Problem Statement

Network reliability represents the statistical probability of a packet being successfully forwarded from one node to the sink. Assume *δ* denotes the QoS level probability that each sensed data packet is successfully received by the sink with a probability not less than *δ*. Let *δ_i_* denote the network reliability of packets successfully transmitted from a node $n_{l}$ with the distance of *i* hops to the sink. 

**Figure 2:**
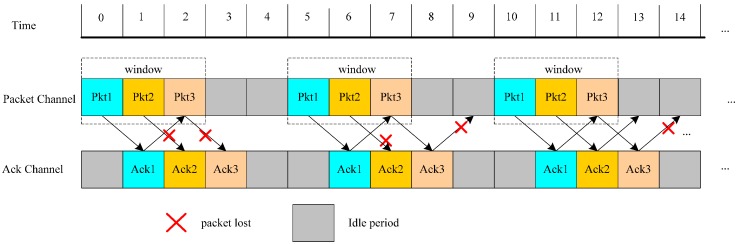
An example of one type of ARQ scheme with the transmission success probability *δ_i_* = 2/3 under *K* = 1 and *W* = 3.

In the paper, we consider the hybrid ARQ scheme for unicast and its operation is as shown in Figure 2. In the scheme, a source node sends data packets continuously with the window size of *W* and it waits for the confirming of ACK of each data. When the sink receives *K* data packets, it will send *K* ACKs for confirmation. After the sending node has received ACKs with the statistical success probability satisfying the transmission constraint *δ_i_*, it would send the subsequent *W* packets. Otherwise, the sending node would retransmit the data packets in the origin window. As shown in Figure 2, the source node first continuously sends three data packets including packet 1, packet 2 and packet 3 in the same window, however, in the transmission process, two packets (packet 2 and packet 3) are lost. Then the source node would then transmit the same three packets, but in the transmission process, packet 2 and ACK 3 are lost. Under this condition, the packet success transmission probability *δ_i_* = 2/3 is also not satisfied. Therefore, the source node transmits the same three packets again. In this transmission process, the third ACK is just lost and accordingly the statistical success transmission probability is 2/3, satisfying the transmission reliability. Then, the source node would send the next three packets (packet 4, packet 5 and packet 6).

As we know that the network lifetime is limited by the node with the maximum energy cost, so we focus on the minimization of the maximum energy consumption among the nodes as well as ensuring the reliability requirements. Consequently, the issue studied in this paper can be abstracted as the following expression:

(2) $$\{\begin{array}{c} \max(T)=\min\underset{0<i\leq n}{\max}(E_{i})\\ \;E_{i}=S_{i}^{1}E_{t}+S_{i}^{2}E_{r}\\ s.t.\;\delta_{i}=\prod_{j\in P_{i}}\beta_{j}>\delta \end{array}$$

The subscripts *i* and *j* represent the hops of nodes to the sink in the network. $S_{i}^{1}$ and $S_{i}^{2}$ respectively represent the amount of sending and receiving data (bits) of node $n_{l}$ with *i* hops to the sink. *E_t_* and *E_r_* denote the energy cost for sending and receiving one bit data. *E_i_* denotes the total energy consumption of sensor $n_{l}$*P_i_* represents the nodes set contained in the routing path from node $n_{l}$ to the destination. *β_j_* indicates the reliability of one hop of the path. 

## 4. An Energy-Aware Hybrid ARQ Scheme

In this section, the overall approach of the proposed energy-aware hybrid ARQ scheme for unicast is presented firstly. Then, the statistical analysis of energy cost of the energy-aware hybrid ARQ scheme operating end-to-end (E2E) and hop-by-hop (HBH) are respectively demonstrated. At the end, we give discussions and analysis on the proposed scheme, including the theory analysis on the energy efficiency and how to select parameters to achieve the optimization goal described by Equation (2).

### 4.1. The Overall Approach

In the scheme, the source node sends data packets continuously with a window size of *W* and it is not necessary to wait for the confirming ACK of each data. When the receiver receives *K* data packets, it will return multiple copies of one confirming ACK to avoid unnecessary data packet retransmissions caused by ACK packet loss, because the ACK packet merely marks the sequence number and its size is much smaller than that of the data packets. When the source node has received ACK packets, it calculates the statistical probability of successful transmission. If the transmission constraint *δ* is satisfied, it would send the subsequent packets with the window size of *W*. Otherwise, the source node would retransmit the data packets in the window. 

**Figure 3:**
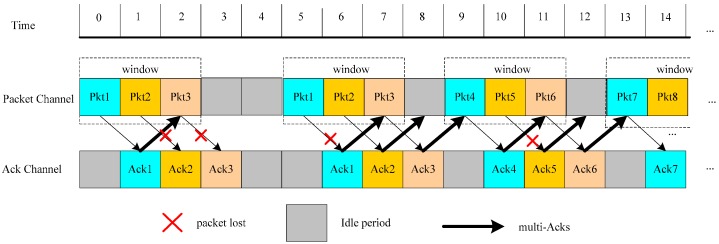
The energy-aware hybrid ARQ scheme with the transmission success probability *δ_i_* = 2/3 under *K* = 1 and *W* = 3.

In the paper, the operation of the proposed energy-aware hybrid ARQ scheme is shown in Figure 3, where the source node continuously sends three data packets including packet 1, packet 2 and packet 3 in the same window firstly. However, in the transmission process, two packets (packet 2 and packet 3) are lost. Then the source node would retransmit the same three packets. But in the transmission process, the packet 1 is lost. Under this condition, the packet successful transmission probability *δ* = 2/3 is satisfied. Then, the source node would send the subsequent three packets (packet 4, packet 5 and packet 6). As we can see that the ACK losses in the transmission process are reduced because multiple copies of ACK are returned for confirming *K* data packets. That improves the ACK transmission reliability and leads to the reduction of data packet retransmission. Thus, the transmission and energy efficiency are further improved by continuously transmitting data packets with a certain window size and returning multiple copies of one ACK for *K* received data packets. The proposed scheme can operate E2E and HBH.

### 4.2. Energy Consumption of Energy-Aware Hybrid E2E ARQ

In this section, we analyze the energy cost when the energy-aware hybrid ARQ scheme operates E2E. In order to do energy consumption analysis, the data load of each node is calculated firstly applying the statistical approach.

**Theorem** **1.** *Assume that* $D_{h,x}^{e2e,t}$ *and* $D_{h,x}^{e2e,r}$ *respectively denote the amount of data that is transmitted and received by node* $n_{l}$ *with* h *hop distance from sink (that is* l=hr+x *(*x<r*)). Meanwhile, it is assumed that* $A_{h,x}^{e2e,t}$ *and* $A_{h,x}^{e2e,r}$ *denote the amount of ACK packet which is transmitted and received by node* $n_{l}$ *respectively. In one round of data gathering, we obtain the following formulas:*

(3) $$D_{h,x}^{e2e,t}=X_{h}^{h}(\delta )+X_{h+1}^{h}(\delta )\frac{l+r}{l}+X_{h+2}^{h}(\delta )\frac{l+2r}{l}+\ldots +X_{h+z}^{h}(\delta )\frac{l+zr}{l}$$

(4) $$D_{h,x}^{e2e,r}=D_{h,x}^{e2e,t}-X_{h}^{h}(\delta )$$

(5) $$A_{h,x}^{e2e,t}=Y_{h}^{h}(\delta )+Y_{h+1}^{h}(\delta )\frac{l+r}{l}+Y_{h+2}^{h}(\delta )\frac{l+2r}{l}+\ldots +Y_{h+z}^{h}(\delta )\frac{l+zr}{l}$$

(6) $$A_{h,x}^{e2e,r}=A_{h,x}^{e2e,t}+Y_{h}^{-1}(\delta )$$

*where, W represents the window size, l = hr + x (x < r) and:*

(7) $$X_{h}^{h}(\delta )=W\cdot \frac{1-{\left(1-\prod_{i=0}^{h-1}{{\bar{p}}_{i}}^{W}{\left(1-{\left(1-\prod_{i=0}^{h-1}{\bar{q}}_{i}\right)}^{n}\right)}^{W/K}\right)}^{L_{h}^{e2e}(\delta )}}{\prod_{i=0}^{h-1}{{\bar{p}}_{i}}^{W}{\left(1-{\left(1-\prod_{i=0}^{h-1}{\bar{q}}_{i}\right)}^{n}\right)}^{W/K}}$$

(8) $$X_{h+j}^{h}(\delta )=W\cdot \frac{1-{\left(1-\prod_{i=0}^{h+j-1}{{\bar{p}}_{i}}^{W}{\left(1-{\left(1-\prod_{i=0}^{h+j-1}{\bar{q}}_{i}\right)}^{n}\right)}^{W/K}\right)}^{L_{h+j}^{e2e}(\delta )}}{\prod_{i=0}^{h+j-1}{{\bar{p}}_{i}}^{W}{\left(1-{\left(1-\prod_{i=0}^{h+j-1}{\bar{q}}_{i}\right)}^{n}\right)}^{W/K}}\prod_{k=h+1}^{h+j}{\bar{p}}_{k}^{W}$$

(9) $$Y_{h+j}^{h}(\delta )=nW\cdot X_{h+j}^{h+j}(\delta )\prod_{k=0}^{h+j-1}{\bar{p}}_{k}^{W}\prod_{k=0}^{h-1}{{\bar{q}}_{k}}^{W/K}/K$$

(10) $$Y_{h}^{h}(\delta )=0$$

(11) $$L_{h}^{e2e}(\delta )=\left\lceil \frac{\log(1-\delta )}{\log(1-\prod_{i=0}^{h-1}{{\bar{p}}_{i}}^{W}{\left(1-{\left(1-\prod_{i=0}^{h-1}{\bar{q}}_{i}\right)}^{n}\right)}^{W/K})}\right\rceil$$

**Proof.**  All packets in a window with size *W* are sent continuously and don’t need to wait for the confirmation of each ACK. In addition, in order to increase the successful probability of receiving ACK, the receiver responses *n* copies of one ACK for each *K* reception of data packet in the proposed protocol. So the probability of all packets are sent successfully in a window is $\prod_{i-0}^{h-1}{\bar{p}}_{i}^{W}{{\left(1-(1-\prod_{i=0}^{h-1}{\bar{q}}_{i}\right)}^{n})}^{W/K}$. Assuming all packets in the window are transmitted with the least attempts *N*, the probability with at least one successful transmission is $1-{{\left(1-\prod_{i-0}^{h-1}{\bar{p}}_{i}^{W}{\left(1-(1-\prod_{i=0}^{h-1}{\bar{q}}_{i}\right)}^{n}\right)}^{W/K})}^{N}$. In order to ensure the statistical reliability of *δ*, we ensure $1-{{\left(1-\prod_{i-0}^{h-1}{\bar{p}}_{i}^{W}{\left(1-(1-\prod_{i=0}^{h-1}{\bar{q}}_{i}\right)}^{n}\right)}^{W/K})}^{N}\geq \delta$ is satisfied. Hence, we can get: $\frac{\log\left(1-\delta \right)}{\log{{\left(1-\prod_{i=0}^{h-1}{\bar{p}}_{i}^{W}(1-(1-\prod_{i=0}^{h-1}{\bar{q}}_{i}\right)}^{n})}^{W/K})}\geq N$ For further processing this expression is rounded up to be an integer to obtain $L_{h}^{e2e}\left(\delta \right),$ which represents the least number of data packet sending times from the source node $n_{l}$ to the sink to ensure the reliability of *δ*. That is:

(12) $$L_{h}^{e2e}(\delta )=\left\lceil \frac{\log(1-\delta )}{\log(1-\prod_{i=0}^{h-1}{{\bar{p}}_{i}}^{W}{\left(1-{\left(1-\prod_{i=0}^{h-1}{\bar{q}}_{i}\right)}^{n}\right)}^{W/K})}\right\rceil$$

Assuming that *X_h_* denotes the times of the window that all data in the window with size *W* are sent successfully with the reliability of *δ*, *X_h_* obeys the upper bound of geometric distribution [20]. That is $X_{h}∼G(L_{h}^{e2e}(\delta ),\prod_{i=0}^{h-1}{{\bar{p}}_{i}}^{W}{\left(1-{\left(1-\prod_{i=0}^{h-1}{\bar{q}}_{i}\right)}^{n}\right)}^{W/K}))$. Therefore, we obtain the following formula:

(13) $$\begin{array}{l} E[X_{h}]=\sum_{k=1}^{L_{h}^{e2e}(\delta )-1}k(\prod_{i=0}^{h-1}{{\bar{p}}_{i}}^{W}{\left(1-{\left(1-\prod_{i=0}^{h-1}{\bar{q}}_{i}\right)}^{n}\right)}^{W/K}){\left(1-\prod_{i=0}^{h-1}{{\bar{p}}_{i}}^{W}{\left(1-{\left(1-\prod_{i=0}^{h-1}{\bar{q}}_{i}\right)}^{n}\right)}^{W/K}\right)}^{k-1}+\ldots \\ \;L_{h}^{e2e}(\delta ){\left(1-\prod_{i=0}^{h-1}{{\bar{p}}_{i}}^{W}{\left(1-{\left(1-\prod_{i=0}^{h-1}{\bar{q}}_{i}\right)}^{n}\right)}^{W/K}\right)}^{L_{h}^{e2e}(\delta )-1}\\ \;=\frac{1-{\left(1-\prod_{i=0}^{h-1}{{\bar{p}}_{i}}^{W}{{\left(1-{\left(1-\prod_{i=0}^{h-1}{\bar{q}}_{i}\right)}^{n}\right)}_{i}}^{W/K}\right)}^{L_{h}^{e2e}(\delta )}}{\prod_{i=0}^{h-1}{{\bar{p}}_{i}}^{W}{\left(1-{\left(1-\prod_{i=0}^{h-1}{\bar{q}}_{i}\right)}^{n}\right)}^{W/K}} \end{array}$$

Considering the window size of *W* and letting $X_{h}^{h}\left(\delta \right)$ represent the statistical amount of data packet sending of the source node $n_{l}$, it is obtained by the following formula:

(14) $$X_{h}^{h}(\delta )=W\cdot \frac{1-{\left(1-\prod_{i=0}^{h-1}{{\bar{p}}_{i}}^{W}{\left(1-{\left(1-\prod_{i=0}^{h-1}{\bar{q}}_{i}\right)}^{n}\right)}^{W/K}\right)}^{L_{h}^{e2e}(\delta )}}{\prod_{i=0}^{h-1}{{\bar{p}}_{i}}^{W}{\left(1-{\left(1-\prod_{i=0}^{h-1}{\bar{q}}_{i}\right)}^{n}\right)}^{W/K}}$$

As for node at (*h* + *j*)*r* + *x* from the sink, the least sending times of data packets and the sending data packet statistics of the node are calculated similarly by the following formulas:

(15) $$L_{h+j}^{e2e}(\delta )=\left\lceil \frac{\log(1-\delta )}{\log(1-\prod_{i=0}^{h+j-1}{{\bar{p}}_{i}}^{W}{\left(1-{\left(1-\prod_{i=0}^{h+j-1}{\bar{q}}_{i}\right)}^{n}\right)}^{W/K})}\right\rceil$$

(16) $$X_{h+j}^{h+j}(\delta )=W\cdot \frac{1-{\left(1-\prod_{i=0}^{h+j-1}{{\bar{p}}_{i}}^{W}{\left(1-{\left(1-\prod_{i=0}^{h+j-1}{\bar{q}}_{i}\right)}^{n}\right)}^{W/K}\right)}^{L_{h+j}^{e2e}(\delta )}}{\prod_{i=0}^{h+j-1}{{\bar{p}}_{i}}^{W}{\left(1-{\left(1-\prod_{i=0}^{h+j-1}{\bar{q}}_{i}\right)}^{n}\right)}^{W/K}}$$

As for node at (*h* + *j*)*r* + *x* from the sink, the data packets transmitted to the node at *hr* + *x* from the sink are:

(17) $$X_{h+j}^{h}(\delta )=W\cdot \frac{1-{\left(1-\prod_{i=0}^{h+j-1}{{\bar{p}}_{i}}^{W}{\left(1-{\left(1-\prod_{i=0}^{h+j-1}{\bar{q}}_{i}\right)}^{n}\right)}^{W/K}\right)}^{L_{h+j}^{e2e}(\delta )}}{\prod_{i=0}^{h+j-1}{{\bar{p}}_{i}}^{W}{\left(1-{\left(1-\prod_{i=0}^{h+j-1}{\bar{q}}_{i}\right)}^{n}\right)}^{W/K}}\prod_{k=h+1}^{h+j}{\bar{p}}_{k}^{W}$$

Since *n* copies of ACK are sent each time when the sink successfully received the *K* data packets, the ACK amount received by the sink from *h* hop is:

(18) $$Y_{h}^{-1}(\delta )=nW\cdot X_{h}^{h}(\delta )\prod_{j=0}^{h-1}{\bar{p}}_{j}^{W}/K$$

At *h* hop, the number of ACK sent for nodes at *h* + *j* can be calculated as follows:

(19) $$Y_{h+j}^{h}(\delta )=nW\cdot X_{h+j}^{h+j}(\delta )\prod_{k=0}^{h+j-1}{\bar{p}}_{k}^{W}\prod_{k=0}^{h-1}{{\bar{q}}_{k}}^{W/K}/K$$

The nodes at *h* hop do not need to send an ACK confirmation for themselves, so:

(20) $$Y_{h}^{h}(\delta )=0$$

**Figure 4:**
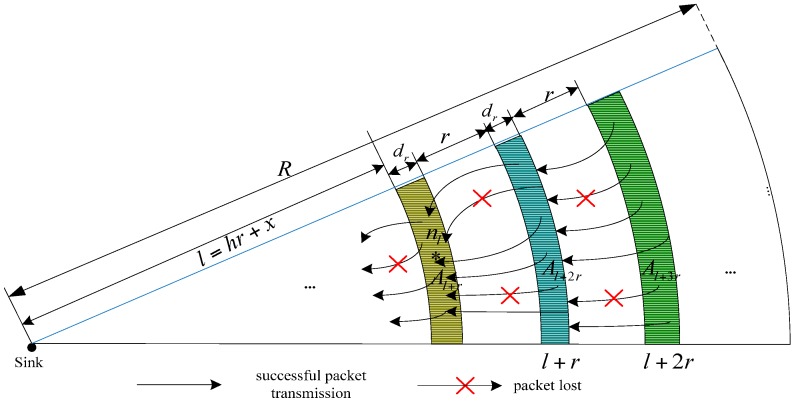
The sketch of data transmission of the node *n_l_*.

Because the nodes at *h* hop need to forward all packets and ACKs which arrive at the *h_th_* hop, as shown in Figure 4, following [18], the total number of transmitted data packets and ACKs of node $n_{l}$ can be computed by:

(21) $$D_{h,x}^{e2e,t}=X_{h}^{h}(\delta )+X_{h+1}^{h}(\delta )\frac{l+r}{l}+X_{h+2}^{h}(\delta )\frac{l+2r}{l}+\ldots +X_{h+z}^{h}(\delta )\frac{l+zr}{l}$$

(22) $$A_{h,x}^{e2e,t}=Y_{h}^{h}(\delta )+Y_{h+1}^{h}(\delta )\frac{l+r}{l}+Y_{h+2}^{h}(\delta )\frac{l+2r}{l}+\ldots +Y_{h+z}^{h}(\delta )\frac{l+zr}{l}$$

where the distance from the node $n_{l}$ to the sink is *l* = *hr* + *x* and *z* is the integer which makes *zr* + *x* just less than *R*.

Considering the process of data transmission without data aggregation, the number of receiving data packets $D_{h,x}^{e2e,r}$ of node $n_{l}$ is equal to the number of sending packets minus its own generated data packets. $A_{h,x}^{e2e,r}$ equals the sum of the number of sent ACKs for other nodes and its own returned ACKs. □

According to the statistical data load of each node and following the typical energy consumption model described in Equation (1), the energy consumption for sending and receiving the data packet can be calculated under the assumption that the number of bits in ACK and data packet are $\lambda_{1}$ and $\lambda_{2}$ respectively and $\lambda_{2}\gg \lambda_{1}$:

(23) $$E_{h,x}^{e2e,t}=\lambda_{2}D_{h,x}^{e2e,t}(E_{elec}+\varepsilon_{\psi }d^{\alpha })$$

(24) $$E_{h,x}^{e2e,r}=\lambda_{2}D_{h,x}^{e2e,r}E_{elec}$$

Similarly, the energy consumption for sending and receiving ACK are calculated by:

(25) $$e_{h,x}^{e2e,t}=\lambda_{1}A_{h,x}^{e2e,t}(E_{elec}+\varepsilon_{\psi }d^{\alpha })$$

(26) $$e_{h,x}^{e2e,r}=\lambda_{1}A_{h,x}^{e2e,r}E_{elec}$$

### 4.3. Energy Consumption of Energy-Aware Hybrid HBH ARQ

In this part, we similarly analyze the energy consumption when the energy-aware hybrid ARQ scheme operates HBH. The data load of each node is analyzed firstly to do energy cost analysis.

**Theorem** **2.** *Assume that* $D_{h,x}^{hbh,t}$ *and* $D_{h,x}^{hbh,r}$ *respectively denote the amount of data that is transmitted and received by node* $n_{l}$ *at* h *hop from sink. Besides, it is assumed that* $A_{h,x}^{hbh,t}$ *and* $A_{h,x}^{hbh,r}$ *denote the amount of ACK data which is transmitted and received by node* $n_{l}$ *respectively. In one round of data gathering, the data load of* $n_{l}$ *is calculated by:*

(27) $$D_{h,x}^{hbh,r}=X_{h+1}^{h+1}(\delta ){{\bar{p}}_{h+1}}^{W}\frac{l+r}{l}+X_{h+2}^{h+1}(\delta ){{\bar{p}}_{h+1}}^{W}\frac{l+2r}{l}+\ldots +X_{h+z}^{h+1}(\delta ){{\bar{p}}_{h+1}}^{W}\frac{l+zr}{l}$$

(28) $$D_{h,x}^{hbh,t}=X_{h}^{h}(\delta )+X_{h+1}^{h}(\delta )\frac{l+r}{l}+X_{h+2}^{h}(\delta )\frac{l+2r}{l}+\ldots +X_{h+z}^{h}(\delta )\frac{l+zr}{l}$$

(29) $$A_{h,x}^{hbh,t}=nD_{h,x}^{hbh,r}/K$$

(30) $$A_{h,x}^{hbh,r}=nD_{h,x}^{hbh,t}{{\bar{p}}_{h}}^{W}{{\bar{q}}_{h-1}}^{W/K}/K$$

where:

(31) $$X_{h}^{h}(\delta )=W\cdot \frac{1-{\left(1-{{\bar{p}}_{h}}^{W}{\left(1-{\left(1-{\bar{q}}_{h}\right)}^{n}\right)}^{W/K}\right)}^{L_{h}^{hbh}(\delta )}}{{{\bar{p}}_{h}}^{W}{\left(1-{\left(1-{\bar{q}}_{h}\right)}^{n}\right)}^{W/K}}$$

(32) $$X_{h+j}^{h}(\delta )=W\cdot \frac{1-{\left(1-{{\bar{p}}_{h+j}}^{W}{\left(1-{\left(1-{\bar{q}}_{h+j}\right)}^{n}\right)}^{W/K}\right)}^{L_{h+j}^{hbh}(\delta )}}{{{\bar{p}}_{h+j}}^{W}{\left(1-{\left(1-{\bar{q}}_{h+j}\right)}^{n}\right)}^{W/K}}$$

(33) $$X_{h+j}^{h}(\delta )=X_{h+j}^{h+1}(\delta ){{\bar{p}}_{h+1}}^{W},\;j\in \left\{1,2,\ldots ,z\right\}$$

**Proof.**  In order to meet the hop transmission reliability $\delta^{1/h}$ and combining theorem 1, the maximum number of the total transmission for nodes at h hop with distance l=hr+x to sink is:

(34) $$L_{h}^{hbh}(\delta )=\left\lceil \frac{\log(1-\delta^{1/h})}{\log(1-{{\bar{p}}_{h}}^{W}{\left(1-{\left(1-{\bar{q}}_{h}\right)}^{n}\right)}^{W/K})}\right\rceil$$

Hence, to ensure the network reliability, the amount of data to be transmitted hop by hop for the nodes is statistically calculated by the following equation:

(35) $$X_{h}^{h}(\delta )=W\cdot \frac{1-{\left(1-{{\bar{p}}_{h}}^{W}{\left(1-{\left(1-{\bar{q}}_{h}\right)}^{n}\right)}^{W/K}\right)}^{L_{h}^{hbh}(\delta )}}{{{\bar{p}}_{h}}^{W}{\left(1-{\left(1-{\bar{q}}_{h}\right)}^{n}\right)}^{W/K}}$$

For a data packet at (h+j)r+x transmitted to the next hop, the expected maximum retransmission number and the amount of data transmitted are respectively computed as follows:

(36) $$L_{h+j}^{hbh}(\delta )=\left\lceil \frac{\log(1-\delta^{1/(h+j)})}{\log(1-{{\bar{p}}_{h+j}}^{W}{\left(1-{\left(1-{\bar{q}}_{h+j}\right)}^{n}\right)}^{W/K})}\right\rceil$$

(37) $$X_{h+j}^{h+j}=X_{h+j}^{h}=W\cdot \frac{1-{\left(1-{{\bar{p}}_{h+j}}^{W}{\left(1-{\left(1-{\bar{q}}_{h+j}\right)}^{n}\right)}^{W/K}\right)}^{L_{h+j}^{hbh}(\delta )}}{{{\bar{p}}_{h+j}}^{W}{\left(1-{\left(1-{\bar{q}}_{h+j}\right)}^{n}\right)}^{W/K}}$$

Therefore, the data load transmitted by node at l=hr+x is calculated by:

(38) $$D_{h,x}^{hbh,t}=X_{h}^{h}(\delta )+X_{h+1}^{h}(\delta )\frac{l+r}{l}+X_{h+2}^{h}(\delta )\frac{l+2r}{l}+\ldots +X_{h+z}^{h}(\delta )\frac{l+zr}{l}$$

In addition, considering the number of received data packets equals the number of data packets sent by the previous node multiplied by the packet loss rate, that is:

(39) $$D_{h,x}^{hbh,r}=D_{h+1,x}^{hbh,t}{{\bar{p}}_{h+1}}^{W},\;j\in \left\{1,2,\ldots ,z\right\}$$

According to the proposed energy-aware hybrid ARQ scheme, the number of the returned ACK equals the number of received data packets divided by K. Moreover, the node does not need to send ACK for its own generated packets, so the nodes at l=hr+x just need to receive data packets and ACKs returned from other nodes. Hence, we obtain the following equations:

(40) $$D_{h,x}^{hbh,r}=X_{h+1}^{h+1}(\delta ){{\bar{p}}_{h+1}}^{W}\frac{l+r}{l}+X_{h+2}^{h+1}(\delta ){{\bar{p}}_{h+1}}^{W}\frac{l+2r}{l}+\ldots +X_{h+z}^{h+1}(\delta ){{\bar{p}}_{h+1}}^{W}\frac{l+zr}{l}$$

(41) $$A_{h,x}^{hbh,t}=nD_{h,x}^{hbh,r}/K$$

As mentioned above, the number of ACK sent for subsequent nodes by node $n_{l}$ is $D_{h,x}^{hbh,t}$. Considering the transmission probability ${\bar{p}}_{h}$ and n copies of ACKs returned for each K data packets with probability ${\bar{q}}_{h-1}$, we can get the amount of ACK data received by node $n_{l}$:

(42) $$A_{h,x}^{hbh,r}=nD_{h,x}^{hbh,t}{{\bar{p}}_{h}}^{W}{{\bar{q}}_{h-1}}^{W/K}/K$$

□

According to the data load of each node and the typical energy consumption model, the energy consumption can be calculated as follows:

(43) $$E_{h,x}^{hbh,t}=\lambda_{2}D_{h,x}^{hbh,t}(E_{elec}+\varepsilon_{\psi }d^{\alpha })$$

(44) $$E_{h,x}^{hbh,r}=\lambda_{2}D_{h,x}^{hbh,r}E_{elec}$$

(45) $$e_{h,x}^{hbh,t}=\lambda_{1}A_{h,x}^{hbh,t}(E_{elec}+\varepsilon_{\psi }d^{\alpha })$$

(46) $$e_{h,x}^{hbh,r}=\lambda_{1}A_{h,x}^{hbh,r}E_{elec}$$

### 4.4. Discussion on the Performance of Energy Efficiency

#### 4.4.1. Performance of Energy Efficiency

In this section, we discuss the conditions under which the proposed ARQ can be more energy efficient than the existing ARQ scheme. In the section, we just do analysis on the E2E operation because the analytical procedure of HBH is similar to that of the E2E operation. As can be seen from the previous sections, the SW-ODMA ARQ protocol is a special case of our proposed energy-aware hybrid ARQ scheme when W = 1 and K = 1. The biggest difference between the proposed energy-aware hybrid ARQ and the original hybrid ARQ scheme is that multiple ACK messages are sent for multiple messages rather than a single message. From the statistical point of view, we need to prove whether the proposed ARQ is more energy efficiency than the original ARQ in one round of data gathering, and the other conclusions are the same for the whole running time of the network.

For the case that multiple ACK messages are sent for a single message, the probability of one returned ACK from the sink to the source node is $\prod_{i=0}^{h-1}{\bar{q}}_{i}$. So the data load of each node is calculated by the following equations:

(47) $${\bar{D}}_{h,x}^{e2e,t}={\bar{X}}_{h}^{h}(\delta )+{\bar{X}}_{h+1}^{h}(\delta )\frac{l+r}{l}+{\bar{X}}_{h+2}^{h}(\delta )\frac{l+2r}{l}+\ldots +{\bar{X}}_{h+z}^{h}(\delta )\frac{l+zr}{l}$$

(48) $${\bar{D}}_{h,x}^{e2e,r}={\bar{D}}_{h,x}^{e2e,t}-{\bar{X}}_{h}^{h}(\delta )$$

(49) $${\bar{A}}_{h,x}^{e2e,t}={\bar{Y}}_{h}^{h}(\delta )+{\bar{Y}}_{h+1}^{h}(\delta )\frac{l+r}{l}+{\bar{Y}}_{h+2}^{h}(\delta )\frac{l+2r}{l}+\ldots +{\bar{Y}}_{h+z}^{h}(\delta )\frac{l+zr}{l}$$

(50) $${\bar{A}}_{h,x}^{e2e,r}={\bar{A}}_{h,x}^{e2e,t}+{\bar{Y}}_{h}^{-1}(\delta )$$

where:

(51) $${\bar{X}}_{h}^{h}(\delta )=W\cdot \frac{1-{\left(1-\prod_{i=0}^{h-1}{{\bar{p}}_{i}}^{W}{\left(1-{\left(1-\prod_{i=0}^{h-1}{\bar{q}}_{i}\right)}^{n}\right)}^{W}\right)}^{{\bar{L}}_{h}^{e2e}(\delta )}}{\prod_{i=0}^{h-1}{{\bar{p}}_{i}}^{W}{\left(1-{\left(1-\prod_{i=0}^{h-1}{\bar{q}}_{i}\right)}^{n}\right)}^{W}}$$

(52) $${\bar{X}}_{h+j}^{h}(\delta )=W\cdot \frac{1-{\left(1-\prod_{i=0}^{h+j-1}{{\bar{p}}_{i}}^{W}{\left(1-{\left(1-\prod_{i=0}^{h+j-1}{\bar{q}}_{i}\right)}^{n}\right)}^{W}\right)}^{{\bar{L}}_{h+j}^{e2e}(\delta )}}{\prod_{i=0}^{h+j-1}{{\bar{p}}_{i}}^{W}{\left(1-{\left(1-\prod_{i=0}^{h+j-1}{\bar{q}}_{i}\right)}^{n}\right)}^{W}}\prod_{k=h+1}^{h+j}{\bar{p}}_{k}^{W}$$

(53) $${\bar{Y}}_{h+j}^{h}(\delta )=nW\cdot {\bar{X}}_{h+j}^{h+j}(\delta )\prod_{k=0}^{h+j-1}{\bar{p}}_{k}^{W}\prod_{k=0}^{h-1}{{\bar{q}}_{k}}^{W}$$

(54) $${\bar{Y}}_{h}^{h}(\delta )=0,\;{\bar{Y}}_{h}^{-1}(\delta )=nW\cdot {\bar{X}}_{h}^{h}(\delta )\prod_{j=0}^{h-1}{\bar{p}}_{j}^{W}$$

(55) $${\bar{L}}_{h}^{e2e}(\delta )=\left\lceil \frac{\log(1-\delta )}{\log(1-\prod_{i=0}^{h-1}{{\bar{p}}_{i}}^{W}{\left(1-{\left(1-\prod_{i=0}^{h-1}{\bar{q}}_{i}\right)}^{n}\right)}^{W})}\right\rceil$$

Therefore, the energy cost is:

(56) $${\bar{E}}_{h,x}^{e2e,t}=\lambda_{2}{\bar{D}}_{h,x}^{e2e,t}(E_{elec}+\varepsilon_{\psi }d^{\alpha })$$

(57) $${\bar{E}}_{h,x}^{e2e,r}=\lambda_{2}{\bar{D}}_{h,x}^{e2e,r}E_{elec}$$

(58) $${\bar{e}}_{h,x}^{e2e.t}=\lambda_{1}{\bar{A}}_{h,x}^{e2e,t}(E_{elec}+\varepsilon_{\psi }d^{\alpha })$$

(59) $${\bar{e}}_{h,x}^{e2e,r}=\lambda_{1}{\bar{A}}_{h,x}^{e2e,r}E_{elec}$$

When satisfying the following condition, the proposed energy-aware hybrid ARQ scheme is more energy efficiency than the typical hybrid ARQ scheme as described in Figure 2:

(60) $$\frac{{\bar{E}}_{h,x}^{e2e,t}+{\bar{E}}_{h,x}^{e2e,r}+{\bar{e}}_{h,x}^{e2e,t}+{\bar{e}}_{h,x}^{e2e,r}}{E_{h,x}^{e2e,t}+E_{h,x}^{e2e,r}+e_{h,x}^{e2e,t}+e_{h,x}^{e2e,r}}>1$$

After the concrete parameters are substituted and further simplified, we can get the following result:

(61) $$\begin{array}{l} \left(1+\frac{E_{elec}}{E_{elec}+\varepsilon_{\psi }d^{\alpha }}\right)\left[\sum_{i=0}^{z}\left({\bar{X}}_{h+i}^{h}(\delta )-X_{h+i}^{h}(\delta )\right)\frac{l+ir}{l}\right]-\frac{E_{elec}}{E_{elec}+\varepsilon_{\psi }d^{\alpha }}\left({\bar{X}}_{h}^{h}(\delta )-X_{h}^{h}(\delta )\right)\\ +\frac{\lambda_{1}}{\lambda_{2}}\left(1+\frac{E_{elec}}{E_{elec}+\varepsilon_{\psi }d^{\alpha }}\right)\left[\sum_{i=0}^{z}W\left({\bar{X}}_{h+i}^{h+i}(\delta )-X_{h+i}^{h+i}(\delta )\right)\prod_{k=0}^{h+i-1}{\bar{p}}_{k}^{W}\prod_{k=0}^{h-1}{{\bar{q}}_{k}}^{W/K}\frac{l+ir}{l}/K\right]\\ +\frac{\lambda_{1}}{\lambda_{2}}\frac{E_{elec}}{E_{elec}+\varepsilon_{\psi }d^{\alpha }}W\left({\bar{X}}_{h}^{h}(\delta )-X_{h}^{h}(\delta )\right)\prod_{k=0}^{h-1}{\bar{p}}_{k}^{W}/K>0 \end{array}$$

Due to the complicated mathematical expressions, the final expression is hard to reduce to one containing parameters W, K, δ, $\lambda_{1}$, $\lambda_{2}$, n, r, ${\bar{p}}_{i}$ and ${\bar{q}}_{i}$. However, from the mathematical expression, we know that the energy efficiency is related with the parameters r, n and K under the given ${\bar{p}}_{i}$, ${\bar{q}}_{i}$, δ and W. Following the similar process, we can discuss and analyze the conditions that the proposed hybrid ARQ scheme can be more effective than the original ARQ operating hop by hop.

#### 4.4.2. Parameter Optimization

According to the discussion above, the energy efficiency is related with the parameters r, n and K under the given ${\bar{p}}_{i}$, ${\bar{q}}_{i}$, δ and W. In this section, how to select parameters of the transmission range of r and the number of returned copies n ACKs for K(K≤W) packets successfully received to achieve the optimization goal of Equation (2) is discussed. It can be seen from the previous sections that the number of data load is different with the distance of source node to the sink, the transmission range of r and the number *n* of returned ACKs for K packets received considering the constraint of required reliability δ. Thus, the energy cost of each node is different with the different parameters. However, the maximum lifetime can be achieved by optimizing the parameters selection under the guarantee of network reliability. The detailed procedure applying the enumeration method to select the optimizing parameters is described in Algorithm 1.

**Algorithm**
**1:** Parameter selection to maximize network lifetime under the required reliability**Input:**
K set {1, …, w}, n set {1,…, m} and r = {r_set}**Output:** the optimized K_min, n_min and r_min1: let globalE = ∞2: for each K in {1, …, w}3: for each n in {1,…, m}4:  for each r = {r_set}5:   for each node with distance l to sink in the network topology6:    calculate the data load under the current r, n and K according to theorem;7:    calculate the energy consumption $E_{l}$ according to Formula 1 and 2;8:    if $E_{l}$ > e_r9:     e_r = $E_{l}$;10:    end if11:   end for12:   if globalE > e_r13:    globalE = e_r;14:    r_min = r;15:    n_min = n;16:    K_min = K;17:   end if18:  end for19: end for20: end for21: output K_min, n_min and r_min

## 5. Performance Evaluation

In this section, the simulation results are presented to evaluate the energy-aware hybrid ARQ scheme under different parameters. The simulation scenario is described firstly, focusing more on the network model and parameters applied in the simulation experiments. Then the evaluation and comparison results are presented to reflect the impact of control parameters on the performance. 

### 5.1. Parameter Settings

**Figure 5:**
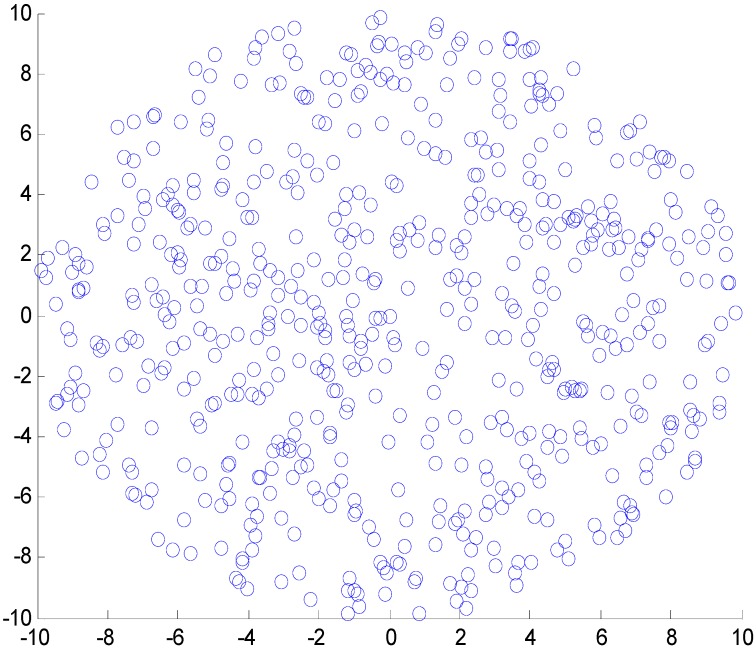
The network topology.

Consider a random flat circle network consisting of 600 sensor nodes that are randomly scattered in a flat network with the radius of 10 (m), which is shown in Figure 5. The sink is located at the center point of the network. The data packet size is fixed $\lambda_{2}$ = 100 bits and the ACK packet size is $\lambda_{1}$ = 20 bits to ensure $\lambda_{2}\gg \lambda_{1}$. The network reliability to be ensured in the following evaluation is randomly set as δ = 0.70. Other parameters of the network are as shown in Table 1. Assuming the window size is W=3. For each node $n_{l}$, the probability of a data and ACK packet successfully transmitted to the next node are respectively assumed $1-p_{i}$ = 0.8 and $1\;-\;q_{i}$ = 0.8 (the value of $p_{i}$ and $q_{i}$ are randomly selected, which do not affect the evaluation on the theoretical analysis on the proposed energy-aware hybrid ARQ scheme). The simulation is conducted to evaluate the correctness and efficiency.

### 5.2. Evaluation on the Energy-Aware Hybrid ARQ Scheme

In the section, the proposed ARQ scheme operating E2E and HBH is evaluated, respectively. In the following evaluations, the relations among energy cost and scheme parameters are described.

#### 5.2.1. Evaluation on the Energy-Aware E2E ARQ

**Figure 6:**
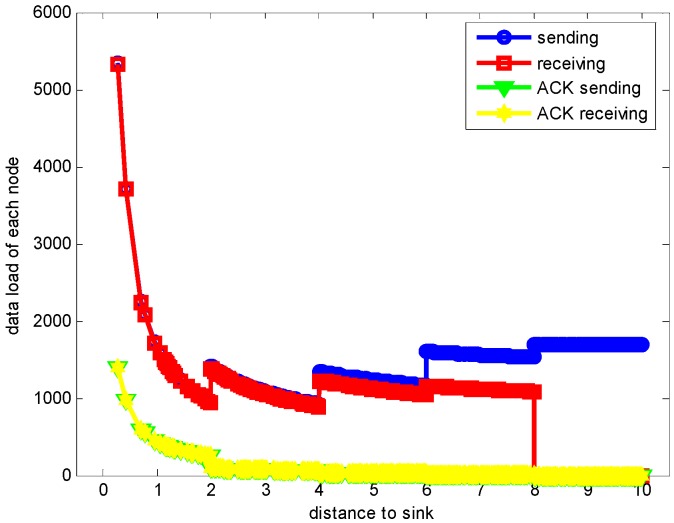
Data load of each node sending and receiving with r=2, n=1, K=1 and δ=0.70.

**Figure 7:**
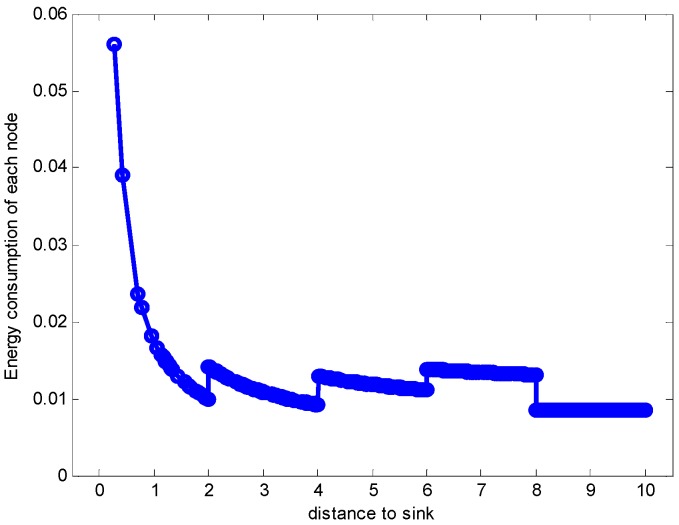
Energy consumption of each node with r=2, n=1, K=1 and δ=0.70.

**Figure 8:**
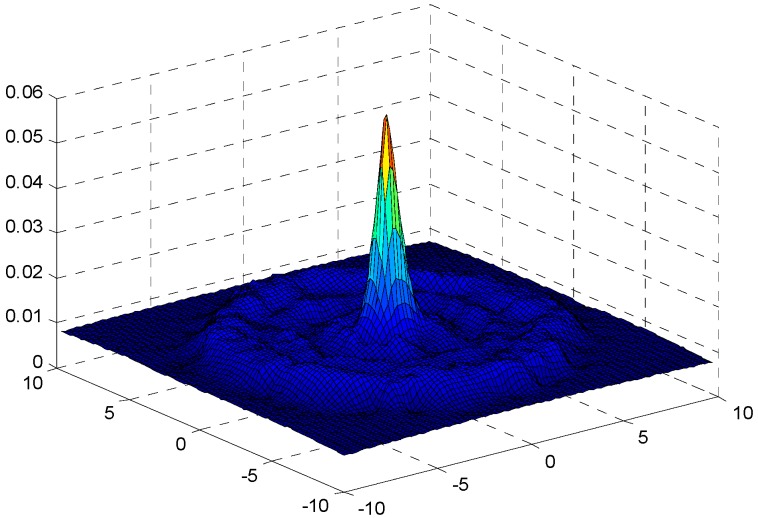
Three dimensional energy consumption diagram with r=2, n=1, K=1 and δ=0.70.

In the part, the evaluation on the proposed hybrid ARQ scheme E2E operation are demonstrated. Figure 6 shows the sending and receiving data load of each node when r=2 m and n=1 under the assurance of the network reliability δ=0.70. From the figure, it can be seen that the data load is increasing as the distance to the sink getting smaller, Figure 7 and Figure 8 describe the energy consumption of each node in two forms. As can be seen from the figures, the nodes closer to the sink bear more data load and have more energy consumption because they not only need to transmit their own data but also forward the data packets from other nodes in the larger part of the network. This is in accordance with the actual analysis result.

In the following, we will evaluate the scheme under different parameters to meet the network reliability δ=0.70. The randomly selected parameters sets are K = {1, 2, 3}, n = {1, 2, 3, 4, 5} and r = {4, 5, 6, 7} (m). For the parameters K and n, their values can only be integers. In the case of W=3, the values of K can only be 1, 2 or 3. When W=1 and K = 1, the SW-ODMA in [18] is the special case of our proposed scheme. For the number of copies of the returned ACKs, n is also with integer values. Hence, in the evaluation n is randomly set {1, 2, 3, 4, 5} in order to gain fully and reliable results. 

**Figure 9:**
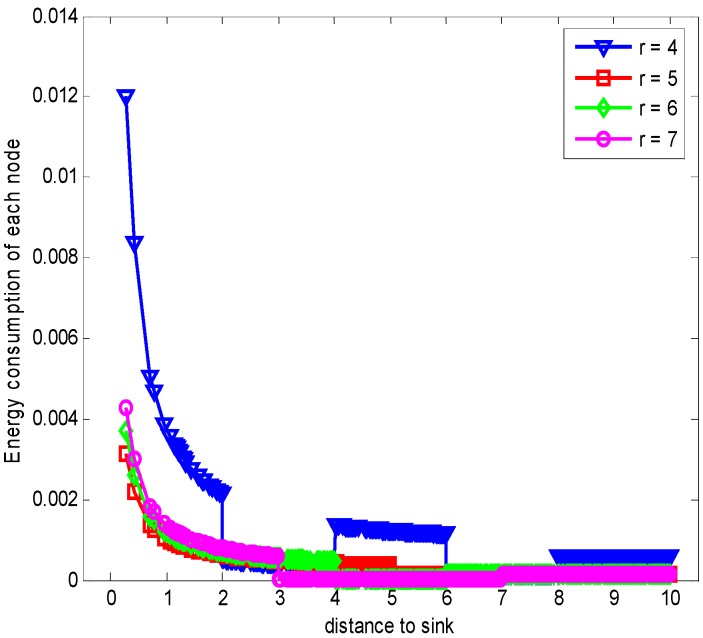
Comparison of energy consumption with different r when K=1, n=1 and δ=0.70.

**Figure 10:**
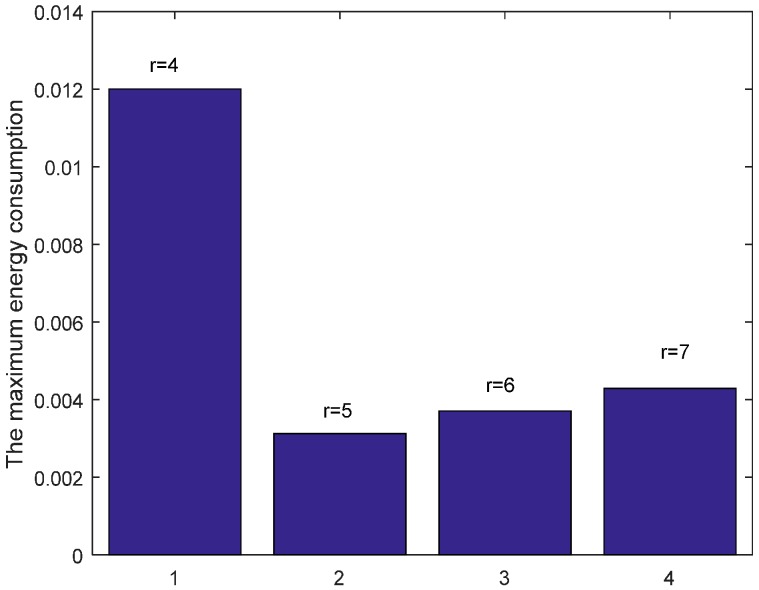
Comparison of the maximum energy consumption with different r when K=1, n=1 and δ=0.70.

When n = 1, it is the original ARQ scheme with one ACK confirmation for one data packet. Besides, to study the influence of the transmission range r in the evaluation, we let the transmission range r is changing and the experimental values are randomly set 4 (m), 5 (m), 6 (m) or 7 (m) according to the network range and assurance of node connectivity. Figure 9 shows the energy consumption of each node with different r when K=1 and n=1. From the figure, we can see that the overall trend of energy consumption is changing bigger for nodes closer to the sink than those nodes far from the sink. Moreover, the energy cost is different with different r values. Especially when r = 5 (m), the maximum node energy consumption is minimized, which can be seen from the Figure 10 obviously. It means that different transmission range r leads to different energy consumption and the energy cost does not have linear relation with r.

**Figure 11:**
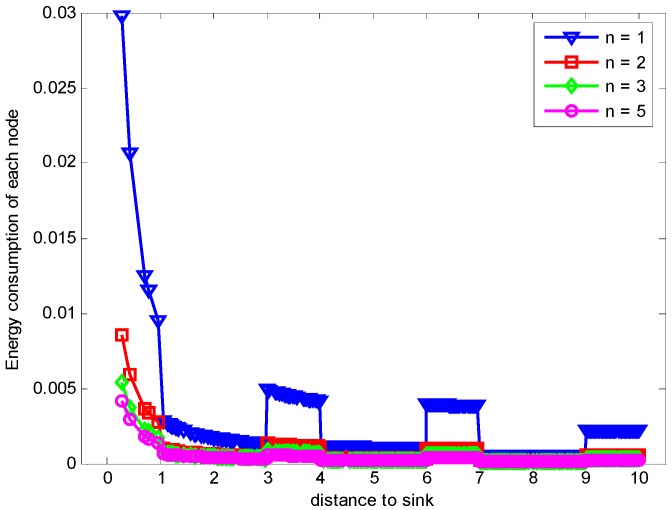
Comparison of energy consumption with different n when K=1, r=3 and δ=0.70.

When the returned ACK has the different number of copies, the data and ACK load under the condition that all nodes generate data for E2E ARQ protocol are different. Figure 11 demonstrates the comparison of energy consumption with different n when K=1 and r=3.

**Figure 12:**
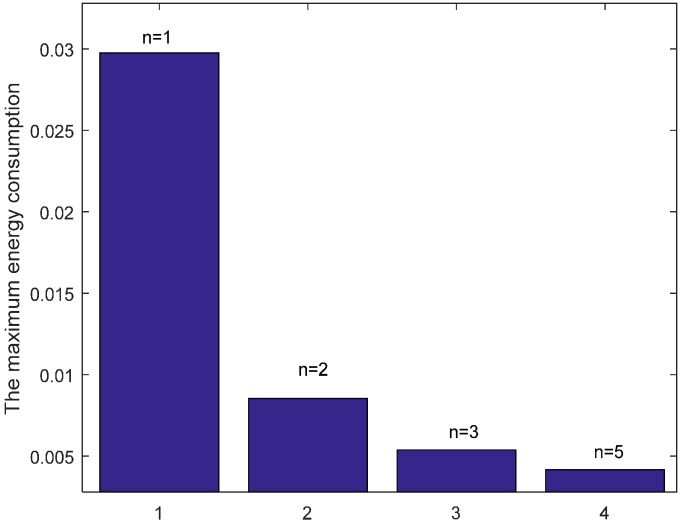
Comparison of the maximum energy consumption with different n when K=1, r=3 and δ=0.70.

From the figure, we can see that the energy consumption can be decreased by reducing data packets retransmission through increasing the number of returned ACK copies. This means that the improvement of the successful reception of ACK by increasing the number of returned ACK copies can reduce the unnecessary retransmission of data packet which consumes more large energy than that of ACK packet. Hence, the total energy consumption of each node is reduced, which leads to the reduction of the maximum energy cost in one data gathering round as shown in Figure 12. The number of returned ACK packets is not the more the better. It needs to satisfy the least number of retransmission times which can be obtained from Theorem 1 for the proposed E2E ARQ protocol. If we continue to increase the value of returned ACK copies, more energy is cost for transmitting the ACKs.

**Figure 13:**
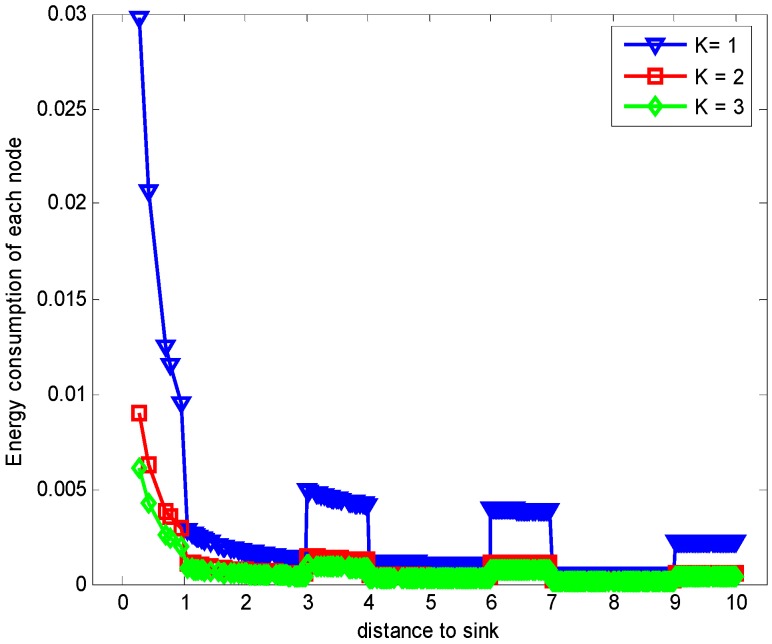
Comparison of energy consumption with different K when n=1, r=3 and δ=0.70.

**Figure 14:**
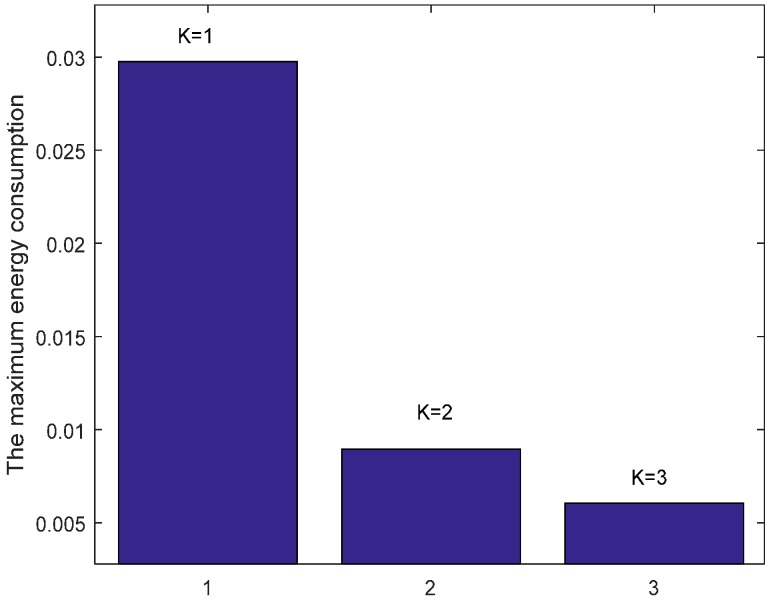
Comparison of the maximum energy consumption with different K when n=1, r=3 and δ=0.70.

Figure 13 shows the comparison of energy consumption with different K when n=1 and r=3. Figure 14 demonstrates the comparison of the maximum energy consumption in the same case. We can see from the figure that the energy consumption is smaller with bigger K under given n, r and δ. This is because that the bigger K leads to smaller number of returned ACKs and so the energy cost is reduced.

#### 5.2.2. Evaluation on the Energy-Aware HBH ARQ

**Figure 15:**
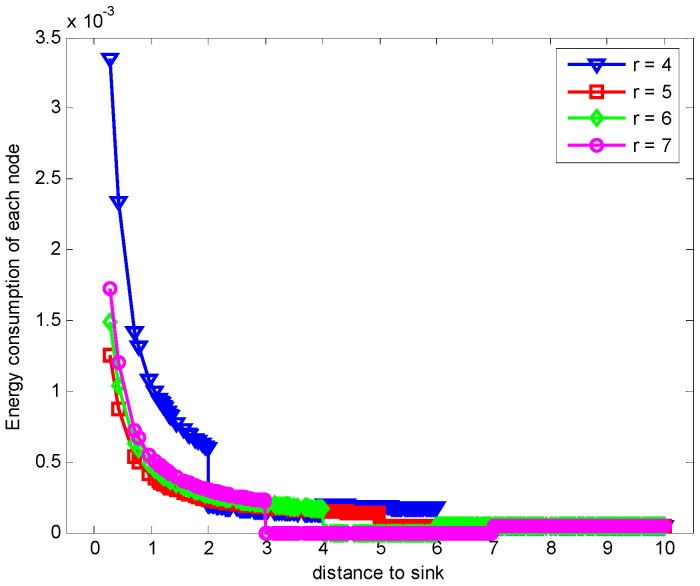
Comparison of energy consumption with different r when K=1, n=1 and δ=0.70.

**Figure 16:**
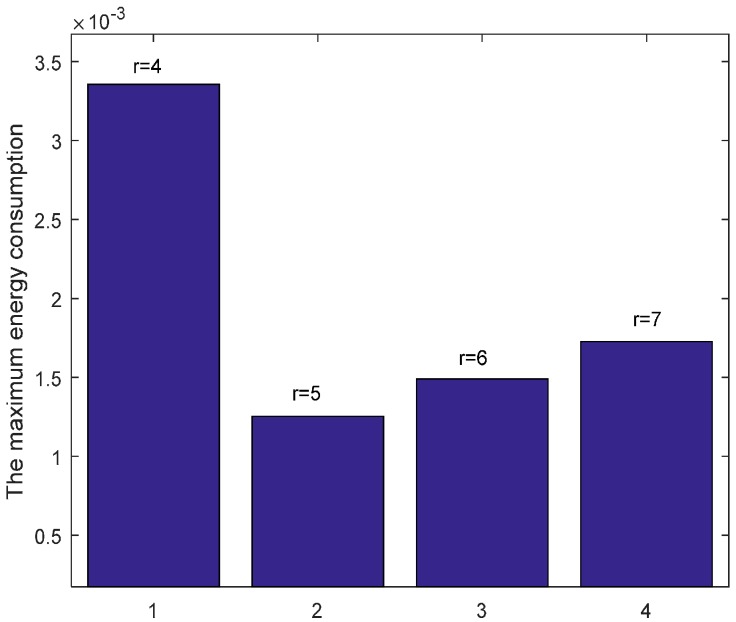
Comparison of the maximum energy consumption with different r when K=1, n=1 and δ=0.70.

**Figure 17:**
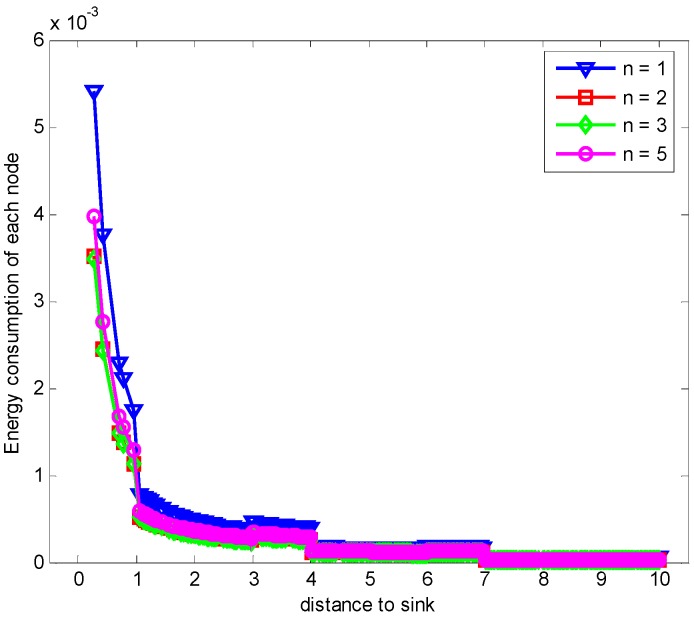
Comparison of energy consumption with different n when K=1, r=3 and δ=0.70.

**Figure 18:**
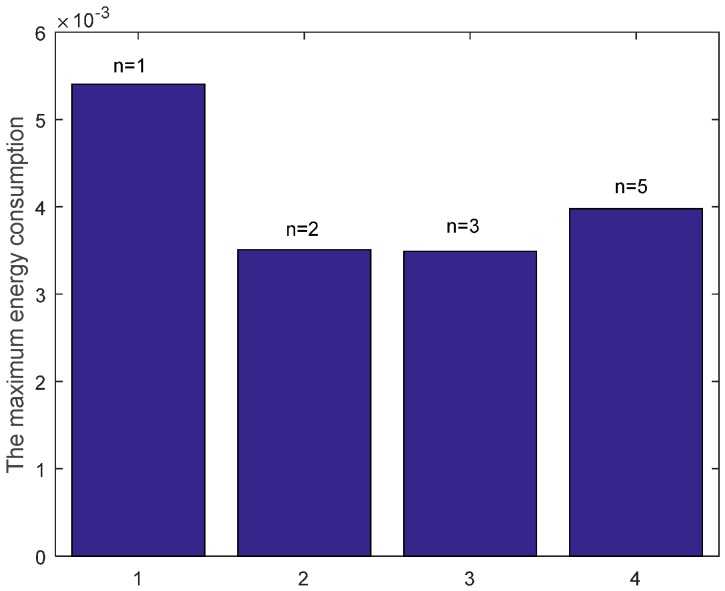
Comparison of the maximum energy consumption with different n when K=1, r=3 and δ=0.70.

**Figure 19:**
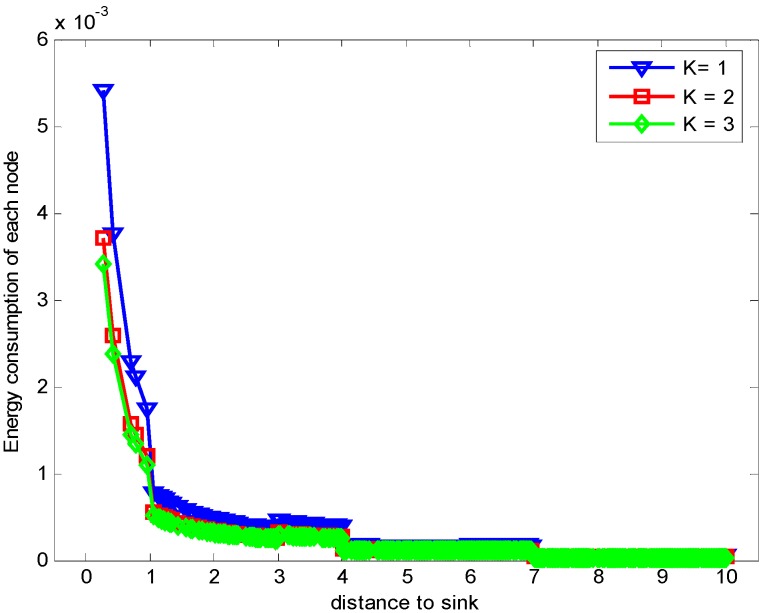
Comparison of energy consumption with different K when n=1, r=3 and δ=0.70.

**Figure 20:**
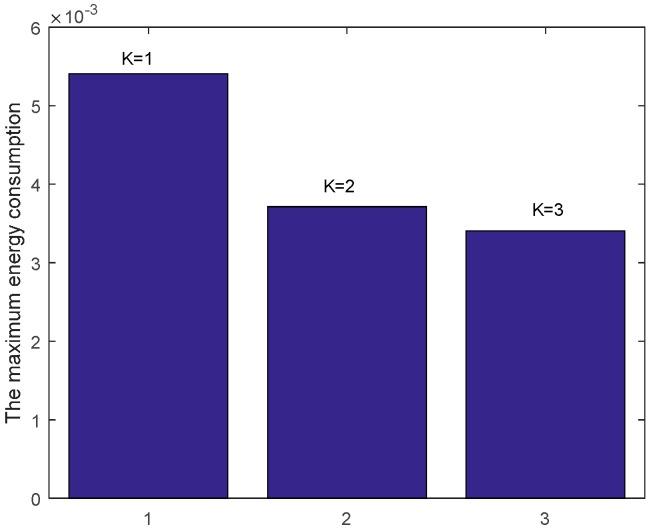
Comparison of the maximum energy consumption with different K when n=1, r=3 and δ=0.70.

In the part, we evaluate the proposed hybrid ARQ scheme operating HBH. Figure 15 presents the comparison of energy consumption with different r when K=1 and n=1, which is the special case of send one data packet and with one ACK packet reply. From the figure, we can see that different r leads to different data load of each node under the constraint of the same network reliability δ=0.70, which causes different node energy consumption. It can be seen from the Figure 15 that there is different energy cost with different r. The maximum energy cost in one round of data gathering is minimized when r = 5 (m) as can be seen from the Figure 16. This means that there is no linear relation between the energy cost and the transmission range. When the returned ACK has different number of copies, the energy consumption of each node is shown in Figure 17. Figure 18 shows the maximum energy cost comparison. As can be seen from the Figure 18, if blindly increasing the number of returned ACK copies, it will cause the waste of energy. This is because the unnecessary increasing of the number of the returned ACK copies leads to more energy cost. Figure 19 shows the energy cost comparison with different K. From the Figure 14, the energy consumption changes with different K values when n=1 and r=3 (m). The ACK load is reduced when the K changes bigger, which can be seen obviously from the Figure 20.

### 5.3. Evaluation on the Parameters Optimization Selection

As demonstrated by the Section 5.2, the energy consumption is related with the parameters r, n and K under the given ${\bar{p}}_{i}$, ${\bar{q}}_{i}$, δ and W parameters, so the optimal selection of the parameters of r, n and K needs to satisfy the optimal energy efficiency goal described by Equation (2). Following the procedure of parameters optimization selection applying the enumeration method, when the predetermined parameters sets are K = {1, 2, 3}, n = {1, 2, 3, 4, 5, 6} and r = {4, 5, 6, 7} (m), the optimized parameters are selected for E2E and HBH operation. 

**Figure 21:**
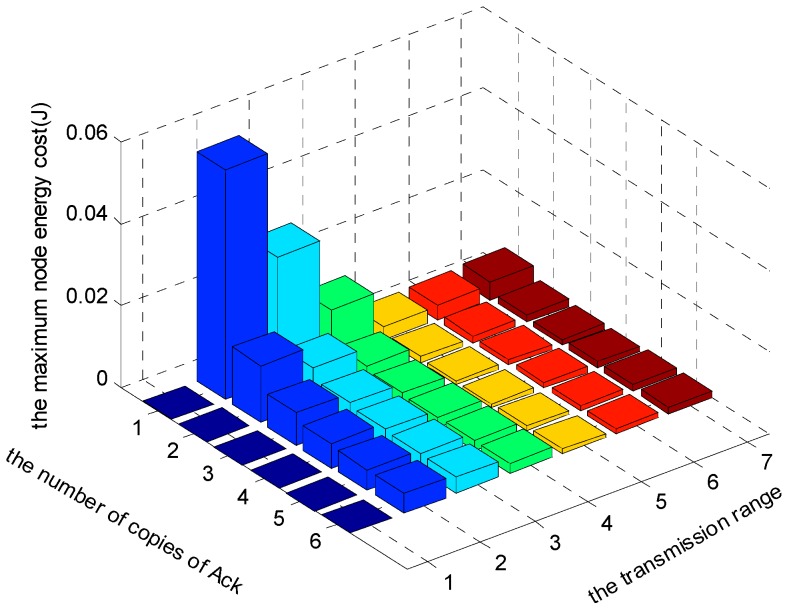
The comparison of maximum energy cost under different r and n when K = 1 and δ=0.70.

**Figure 22:**
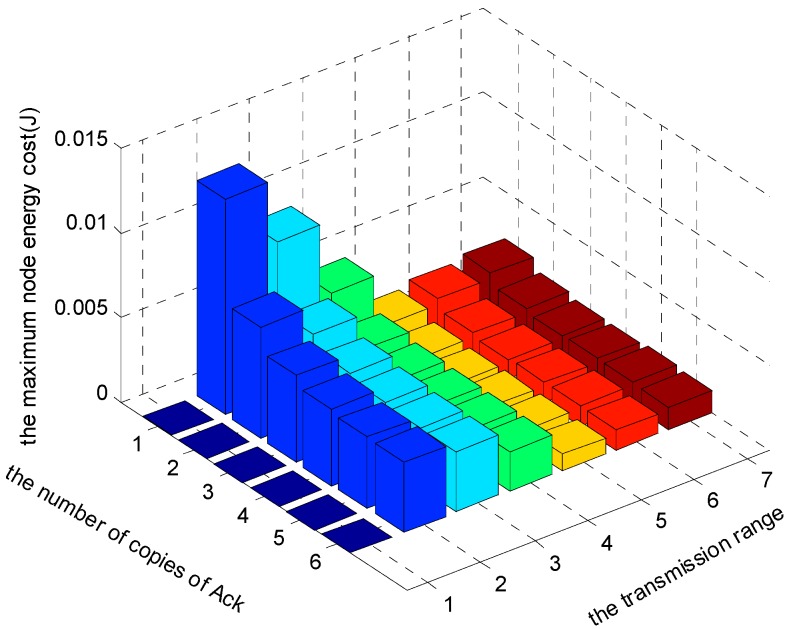
The comparison of maximum energy cost under different r and n when K = 2 and δ=0.70.

**Figure 23:**
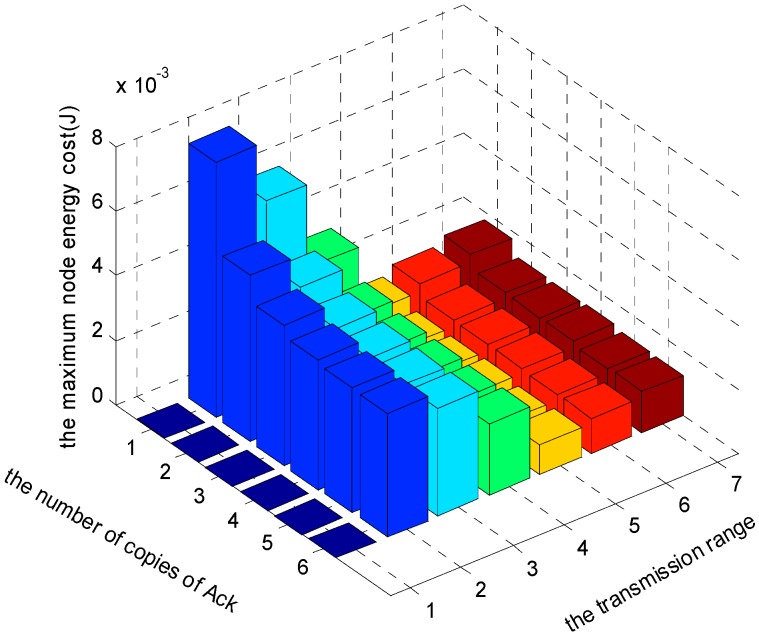
The comparison of maximum energy cost under different r and n when K = 3 and δ=0.70.

Figure 21, Figure 22 and Figure 23 show the comparison of maximum energy cost under different r, n and K in one round of data gathering for E2E operation. As can be seen from the figures, when r = 5 (m), n = 5 and K = 3 are selected from the set of r = {2, 3, 4, 5, 6, 7} (m), n = {1, 2, 3, 4, 5, 6} and K = {1, 2, 3}, the maximum energy cost in one round of data gathering is minimized.

**Figure 24:**
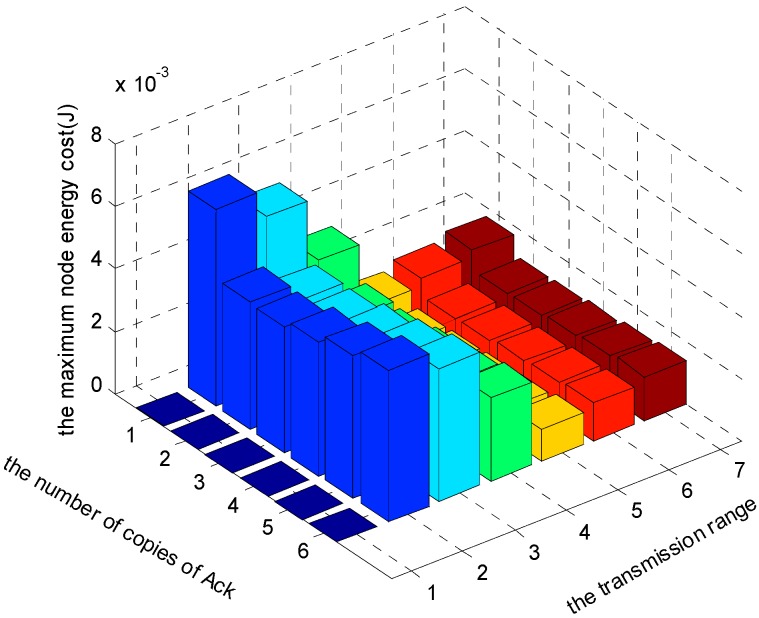
The comparison of maximum energy cost under different r and n when K = 1 and δ=0.70.

**Figure 25:**
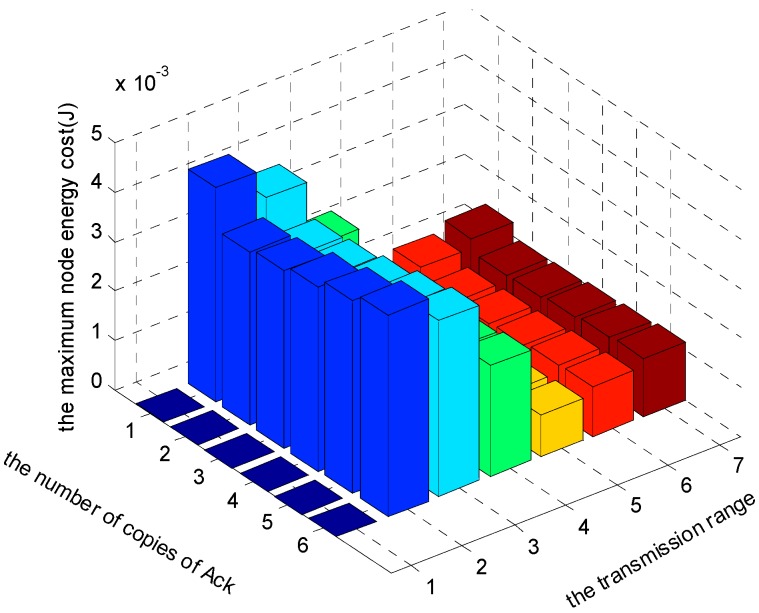
The comparison of maximum energy cost under different r and n when K = 2 and δ=0.70.

**Figure 26:**
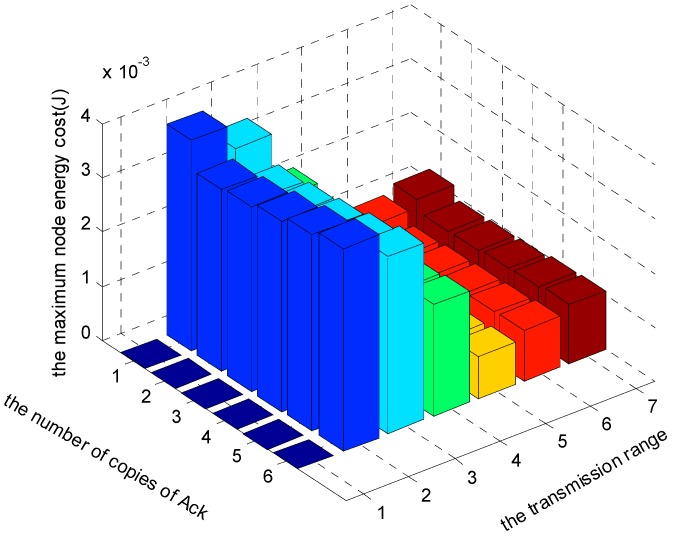
The comparison of maximum energy cost under different r and n when K = 3 and δ=0.70.

Figure 24, Figure 25 and Figure 26 show the comparison of maximum energy cost under different r, n and K in one round of data gathering for HBH operation. As can be seen from the figures, when r = 5 (m), n = 2 and K = 3 are selected from the set of r = {2, 3, 4, 5, 6, 7} (m), n = {1, 2, 3, 4, 5, 6} and K = {1, 2, 3}, the maximum energy cost in one round of data gathering is minimized.

### 5.4. Evaluation on the Performance by Comparison with SW-ODMA ARQ 

**Figure 27:**
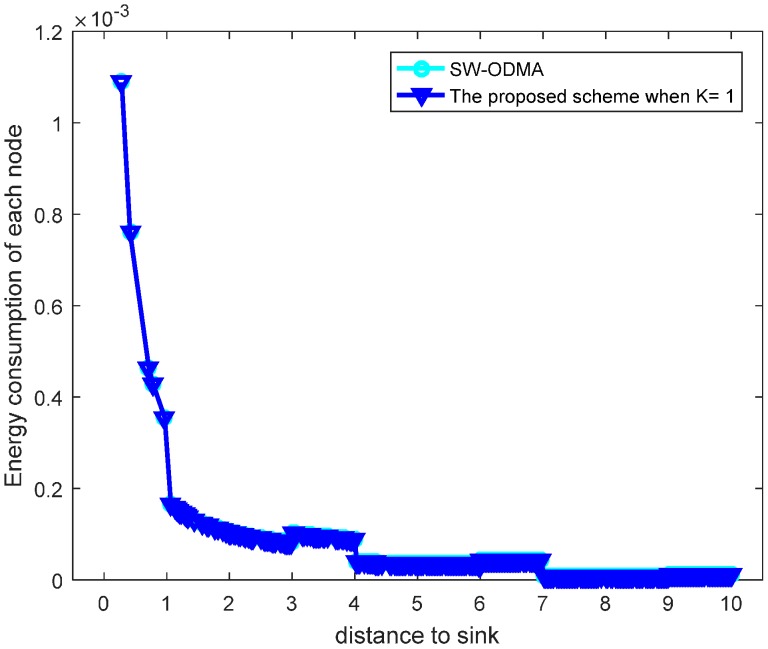
Comparison of energy consumption with SW-ODMA under W=1, K = 1, n=2, r=3 and δ=0.70.

**Figure 28:**
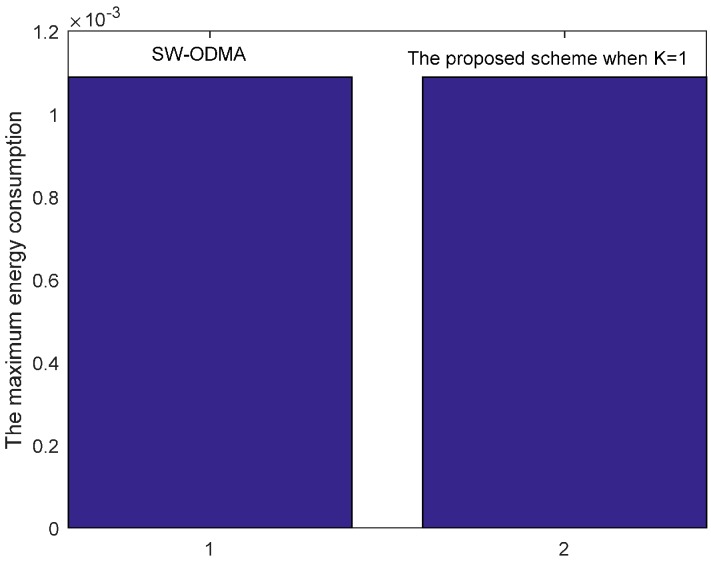
Comparison of the maximum energy consumption with SW-ODMA under W=1, K = 1, n=2, r=3 and δ=0.70.

**Figure 29:**
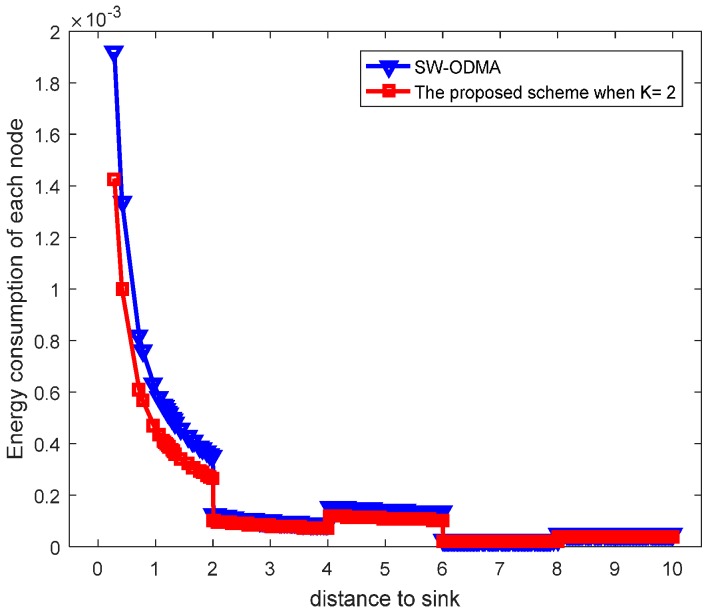
Comparison of energy consumption between scheme following SW-ODMA and the proposed scheme when W=2, n=2, r=4 and δ=0.70.

Our proposed scheme is a generalization of the original SW-ODMA ARQ protocol [18]. The SW-ODMA ARQ scheme is the special case of the proposed scheme when the sending widow size W=1 and K = 1. In this case, the energy consumption is the same as shown in Figure 27 and Figure 28 when operating E2E and with the assurance of the same network reliability δ=0.70. For E2E operation, when the sending window size W=2 and the sending packets in the window are transmitted continuously, the comparison of energy consumption between scheme following SW-ODMA with multiple of one ACK for one receiving data packet and the proposed hybrid scheme when K = 2 is shown in Figure 29 for one round of data gathering. In the comparison, the parameters of the number of the returned ACK and the transmission range are randomly selected n=2 and r=4 with the constraint of the network reliability δ=0.70.

**Figure 30:**
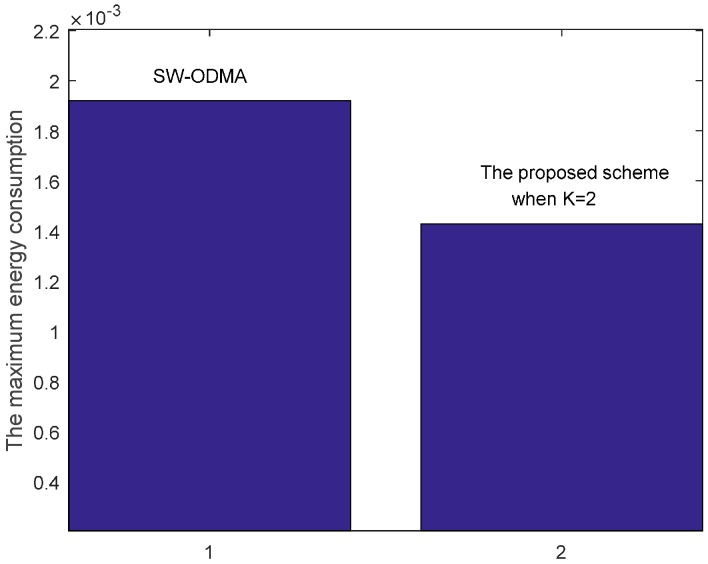
Comparison of the maximum energy consumption between scheme following SW-ODMA and the proposed scheme when W=2, n=2, r=4 and δ=0.70.

**Figure 31:**
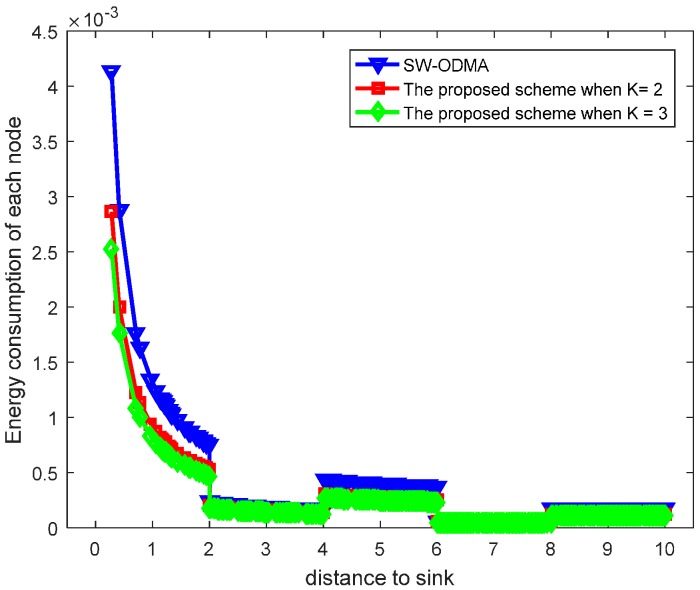
Comparison of energy consumption between scheme following SW-ODMA and the proposed scheme when W=3, n=2, r=4 and δ=0.70.

Figure 30 shows the maximum energy cost comparison. As can be seen from the figure, the maximum energy cost is reduced by 25.60%. When the sending window size W=3, the comparison of energy consumption following SW-ODMA with multiple of one ACK for one receiving data packet and the proposed hybrid scheme when K = 2 and K = 3 is shown in Figure 31. 

**Figure 32:**
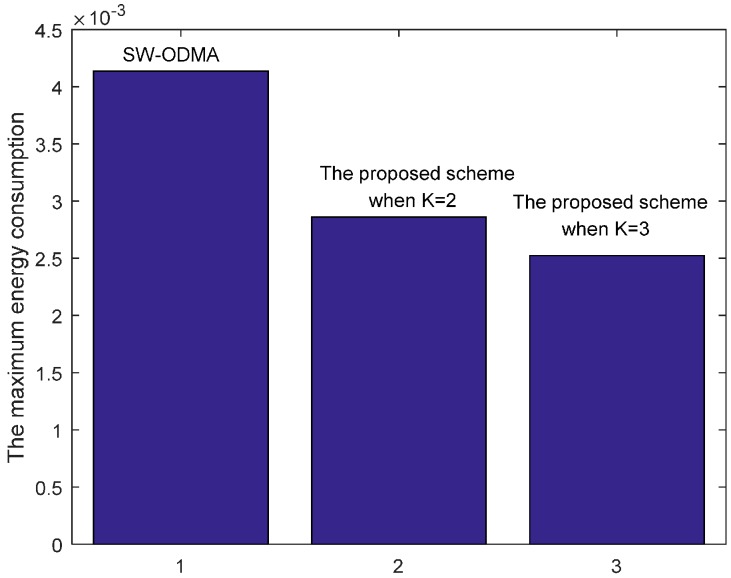
Comparison of the maximum energy consumption between scheme following SW-ODMA and the proposed scheme when W=3, n=2, r=4 and δ=0.70.

Figure 32 shows the maximum energy cost comparison. As can be seen from the figure, the maximum energy cost is reduced by 30.85% when K = 2 and by 38.99% when K = 3 respectively.

**Figure 33:**
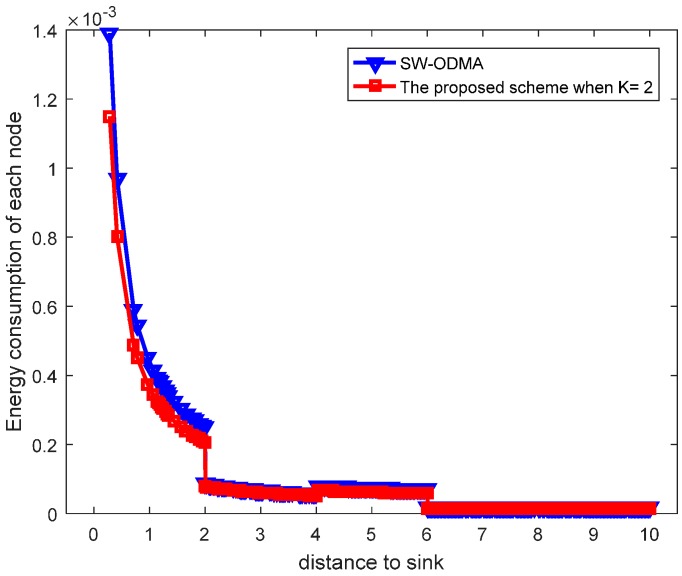
Comparison of energy consumption between scheme following SW-ODMA and the proposed scheme when W=2, n=2, r=4 and δ=0.70.

**Figure 34:**
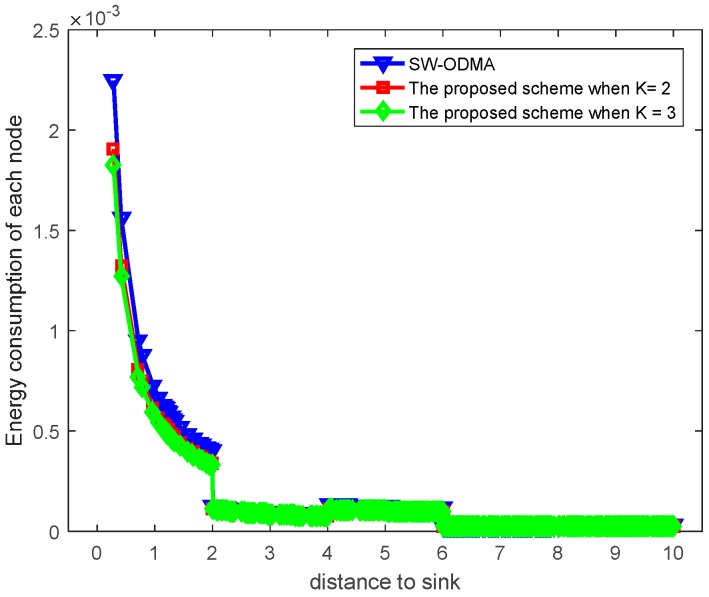
Comparison of energy consumption between scheme following SW-ODMA and the proposed scheme when W=3, n=2, r=4 and δ=0.70.

**Figure 35:**
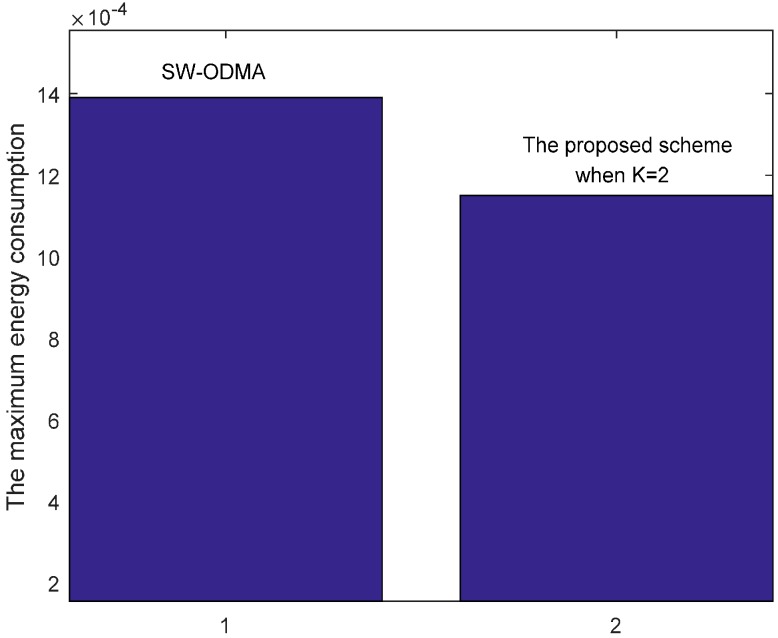
Comparison of the maximum energy consumption between scheme following SW-ODMA and the proposed scheme when W=2, n=2, r=4 and δ=0.70.

**Figure 36:**
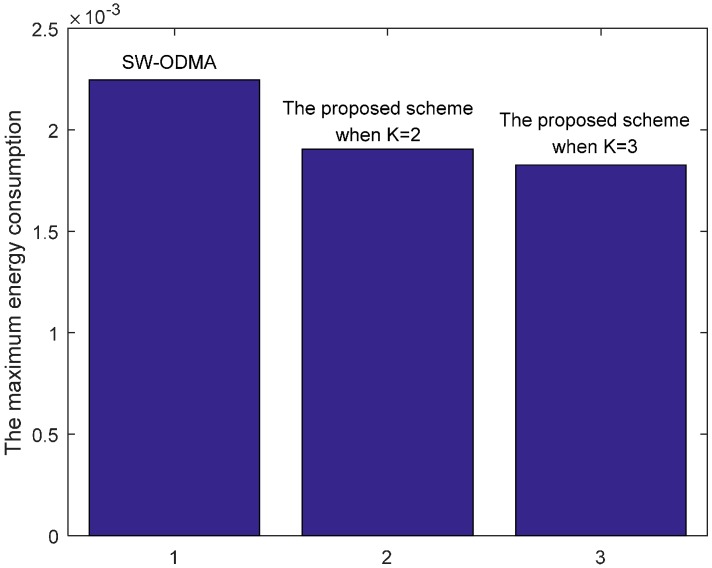
Comparison of the maximum energy consumption between scheme following SW-ODMA and the proposed scheme when W=3, n=2, r=4 and δ=0.70.

Comparing the proposed scheme with the scheme following SW-ODMA operating HBH, Figure 33 and Figure 34 respectively show the energy cost comparison of each node when the sending window size W=2 and W=3 under randomly selected n=2, r=4 and the same network reliability δ=0.70. Figure 35 and Figure 36 respectively demonstrate the maximum energy cost comparison under the cases. From Figure 35, we can see that the maximum energy cost is reduced by 17.30%. Besides, the maximum energy cost is reduced by 15.20% when K = 2 and by 18.67% when K = 3 respectively as can be seen from the Figure 36.

## 6. Conclusions

In this paper, the problem of an effective data transmission scheme to reduce energy cost and ensure transmission reliability with hybrid ARQ is studied. Data transmission should take reliability as well as energy efficiency into consideration. To address these problems, an energy-aware hybrid ARQ scheme is proposed to improve the energy efficiency with the assurance of network transmission reliability. The theoretical analysis of data load of each node with reliability constraint for flat circle network is presented. Based on this, the energy efficiency is discussed. Because the energy efficiency is related with the parameters r, n and K under the given ${\bar{p}}_{i}$, ${\bar{q}}_{i}$, δ and W we study how to select parameters applying the enumeration method to obtain the optimal energy efficiency goal. The simulations are extensively done to evaluate the theoretical analysis and verify the effectiveness of the proposed ARQ scheme.

## Figures and Tables

**Table 1:** Parameters and corresponding values.

Parameter (units)	Value
Threshold distance (*d*_0_) (m)	87
Sensing range *r* (m)	15
*E_elec_* (nJ/bit)	50
*ε_fs_* (pJ/bit/m^2^)	10
*ε_amp_* (pJ/bit/m^4^)	0.0013
